# PTPN22 Dephosphorylates CBL to Inhibit PD-L1 Ubiquitination and Drive Immunosuppression in Renal Cell Carcinoma

**DOI:** 10.7150/ijbs.122418

**Published:** 2026-01-01

**Authors:** Taian Jin, Jiahui Ma, Luping Wang, Xinbo Liu, Mengting Wu, Binqi Wang, Chan Gao, Siqi Zhu, Ruikai Zhang, Fanwei Xia, Jingkui Tian, Wei Zhu, Juan Jin, Qiang He

**Affiliations:** 1 Department of Nephrology, the First Affiliated Hospital of Zhejiang Chinese Medical University (Zhejiang Provincial Hospital of Chinese Medicine), Hangzhou 310006, Zhejiang, P.R. China.; 2 Zhejiang Key Laboratory of Research and Translation for Kidney Deficiency-Stasis-Turbidity Disease; Zhejiang-Macau International Joint Laboratory of Integrated Traditional Chinese and Western Medicine for Nephrology and Immunology, Hangzhou 310006, Zhejiang, P.R. China.; 3 Hangzhou Institute of Medicine (HIM), Chinese Academy of Sciences, Hangzhou 310006, Zhejiang, P.R. China.; 4 Affiliated Hangzhou First People's Hospital, School of Medicine, Westlake University, Hangzhou 310006, Zhejiang, P.R. China.

**Keywords:** PTPN22, CBL, PD-L1, Dephosphorylation, Ubiquitination, renal cell carcinoma

## Abstract

High lymphocyte infiltration and T cell exhaustion characterize the tumor microenvironment in renal cell carcinoma (RCC). Protein tyrosine phosphatase N22 (PTPN22), a protein tyrosine phosphatase that mediates proteins tyrosine dephosphorylation, is a negative regulator of T cell receptor signaling, but its role in tumor cells has been underappreciated. PTPN22 is highly expressed in RCC cells and positively correlated with PD-L1 protein expression. CBL was newly identified as a substrate of PTPN22, and our study reveals for the first time that CBL mediates the K48-linked ubiquitination of PD-L1. PTPN22 specifically interacts with CBL, catalyzing the dephosphorylation of tyrosine 700 and inhibiting CBL binding to PD-L1, thereby preventing CBL-mediated ubiquitination and degradation of PD-L1. This stabilization of PD-L1 promotes T cell exhaustion and immunosuppression. Through screening of traditional Chinese medicine monomers, we identified curcumin as a potential PTPN22 inhibitor. Curcumin reduces PTPN22 stability and PTPN22 expression by directly binding to PTPN22. *In vivo* experiments demonstrated that combining curcumin with immune checkpoint inhibition (ICIs) further promotes T cell activation, inhibits Tregs infiltration, and enhances ICIs efficacy against tumor growth. Therefore, PTPN22 represents a therapeutic target for improving T cell exhaustion in RCC and enhance ICIs efficacy through CBL-mediated ubiquitination and degradation of PD-L1.

## 1. Introduction

Renal cell carcinoma (RCC) originates from the epithelial cells of the renal tubules and ranks among the ten most prevalent malignant tumors 1. While surgical remains the optimal treatment for early-stage RCC, approximately 30% of patients present with metastatic disease at initial diagnosis 2. Given RCC's resistance to radiotherapy and chemotherapy, targeted therapy combined with immunotherapy is considered the first-line treatment for unresectable RCC 3. However, intrinsic or acquired resistance to immune checkpoint inhibition (ICI) therapies limits the durable response in most RCC patients 4. Consequently, identifying potential drivers of immune evasion in RCC and developing new strategies to modulate immune checkpoint expression to enhance sensitivity to ICI therapy is of utmost imperative.

RCC is characterized by significant immune infiltration, which encompasses a diverse array of immune cell types 5. Although RCC is amenable to immunotherapy, its tumor microenvironment (TME) exhibits unique immunological features compared to other tumor types 6. In RCC, baseline CD8+ T cell infiltration is paradoxically associated with poorer prognosis. A comprehensive mass cytometry analysis of primary clear cell Renal cell carcinoma (ccRCC) revealed extensive infiltration by CD8+ PD-1+ T cells, with markers of T cell exhaustion correlating with survival outcomes 7. These findings underscore the prevalence of immune escape in RCC and its critical role in long-term survival. PD-L1 (CD274) binds to PD-1, resulting in exhaustion of CD8+ cytotoxic T cells that have entered the tumor. The interaction disrupts the tumor-reactive lymphocyte response and functions as a suppressor of regulatory T cells (Tregs), thereby inhibiting immune activation and evading immune surveillance 8, 9. Therefore, blocking the PD-1/PD-L1 axis is considered a promising strategy to restore T cell function.

PD-L1 expression is regulated by multiple factors. Tumor-specific PD-L1 upregulation may be driven by pro-survival pathways such as MAPK and PI3K/Akt, as well as transcription factors such as HIF-1, STAT3 and NF-ĸB 10-12. Additionally, numerous studies indicate that PD-L1 is also regulated by various protein post-translational modification. These modifications include ubiquitination, glycosylation, phosphorylation, palmitoylation, and acetylation, which together intricately modulate PD-L1 expression 13-15. The ubiquitin-proteasome system serves as a critical mechanism for intracellular protein degradation 16. E3 ubiquitin ligases are regulated by modifications including phosphorylation, methylation, and acetylation, which can promote or inhibit substrate degradation 17, 18. Phosphorylation exhibits significant crosstalk with ubiquitination, widely affecting the interaction between receptors and substrates 19, 20. c-CBL (CBL), a member of the Casitas B lineage lymphoma (CBL) protein family, negatively regulates receptor tyrosine kinases (RTKs) through ubiquitination and degrades various proteins targets 21. This protein contains a RING finger domain that functions as an E3 ubiquitin ligase, mediating ubiquitination of its targets. The C terminus features a proline-rich domain responsible for protein-protein interactions. The phosphorylation levels of the three exposed tyrosines (Tyr700, Tyr731, and Tyr744) in this region modulate CBL's interactions with other proteins 22.

PTPN22 belongs to a gene family encoding protein tyrosine phosphatase (PTPs). Studies demonstrate that PTPN22 potently inhibits T cell activation through inhibitory dephosphorylation of key signal transduction mediators downstream of the T cell antigen receptor (TCR) 23. Both biological knockout and pharmacological inhibition of PTPN22 reduces tumor growth and have the potential to synergistically enhance tumor clearance when combined with PD-L1 blockers 24. However, research on PTPN22 has primarily focus on autoimmune diseases and the immune system, with limited studies examining its role in tumor cells. In this study, we present evidence that PTPN22 interacts with CBL and increases PD-L1 stability. PTPN22 inhibits the ligase activity of CBL through dephosphorylation, protecting PD-L1 from ubiquitination, as well as proteasomal degradation in RCC cells. Additionally, we identified curcumin (Cur), a traditional Chinese medicine monomer, as a promising candidate as a PTPN22 inhibitor. Curcumin co-treatment with immune checkpoint inhibitors (ICIs) inhibited RCC growth enhanced the tumor microenvironment.

## 2. Results

### 2.1 PTPN22 is upregulated in RCC cells and positively correlates with PD-L1

**Figure 1:**
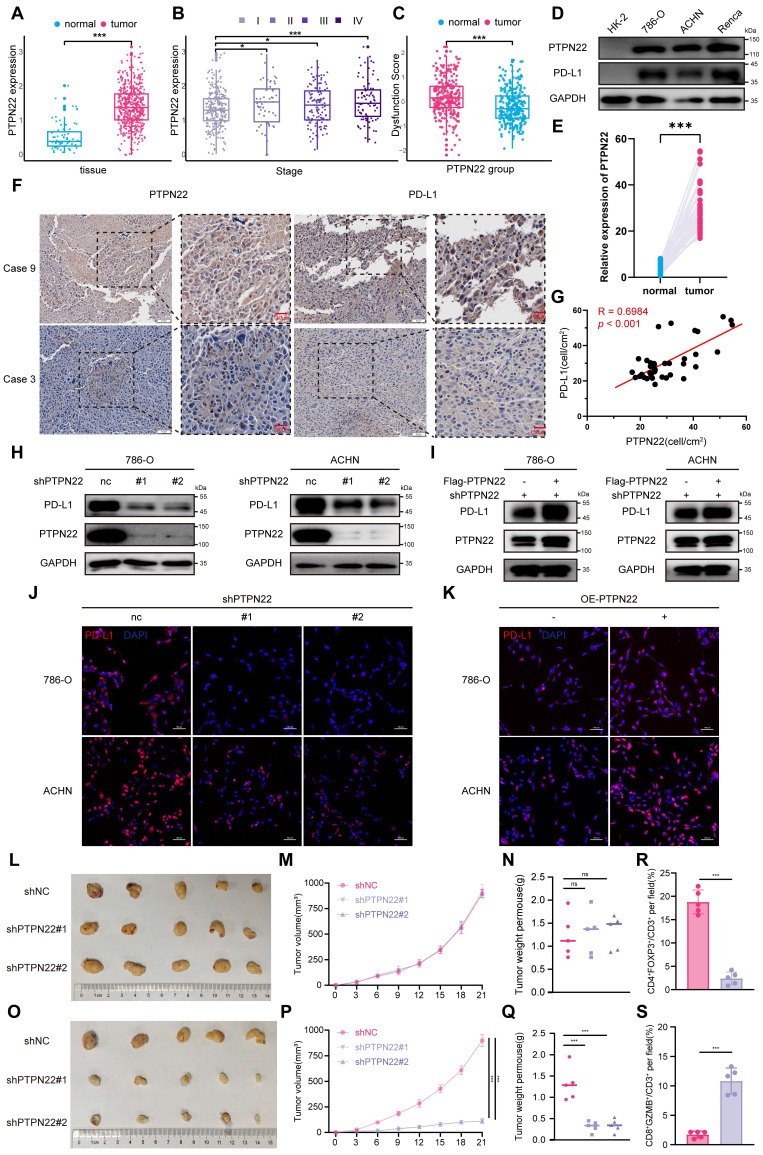
PTPN22 is upregulated in RCC and positively correlates with PD-L1. A) PTPN22 mRNA levels in ccRCC vs. normal tissues from TCGA database. B) PTPN22 mRNA levels across different ccRCC stages from TCGA database. C) TIDE analysis showing positive correlation between PTPN22 and immune dysfunction score in TCGA dataset. D) Representative western blot of PTPN22 and PD-L1 protein levels in the immortalized HK-2 renal epithelial cell line and RCC cell lines (786-O, ACHN, and Renca), with GAPDH as loading control. E) Positive correlation between PTPN22 and PD-L1 mRNA in TCGA dataset. F) Representative immunohistochemistry (IHC) images of PTPN22 and PD-L1 expression in patient-derived tissues. G) Correlation analysis of PTPN22 and PD-L1 protein levels in patient-derived tissues. H) Representative western blot of PTPN22 and PD-L1 protein levels in 786-O and ACHN cells after PTPN22 knockdown. I) Representative Western blot of PTPN22 and PD-L1 protein levels in 786-O and ACHN cells after a rescue experiment following PTPN22 knockdown. J) Representative Immunofluorescence (IF) images of PD-L1(red) in 786-O and ACHN cells after PTPN22 knockdown. Scale bar = 100 μm. K) Representative IF images of PD-L1(red) in 786-O and ACHN cells after PTPN22 overexpression. Scale bar = 100 μm. L-N) Representative images (L), growth curves (M) and tumor weight (N) of BALB/c nude mice with indicated treatment. (n = 5 per group). O-Q) Representative images (O), growth curves (P) and tumor weight (Q) of BALB/c mice with indicated treatment. (n = 5 per group). R, S) Statistical analysis of the proportion of CD4+FOXP3+ (R) and CD8+GZMB+ (S) cells among CD3+ tumor-infiltrating lymphocytes in different groups. ns, not significant; *p < 0.05, **p < 0.01 and ***p < 0.001.

Numerous studies have demonstrated that PTPN22 modulates the immune response to tumor therapy through modulation of T cell activation 25, 26. To further investigate the potential role of PTPN22 in tumor cells, we systematically evaluated PTPN22 expression levels in tumor and normal tissues utilizing the Cancer Genome Atlas (TCGA) database. Bioinformatic analyses revealed that PTPN22 was significantly upregulated in kidney renal clear cell carcinoma (KIRC) (**Figure 1**A, **S1**A). Moreover, the analysis of PTPN22 expression at different stages demonstrated a progressive elevation with disease progression (**Figure 1**B). PTPN22 not only altered the composition of immune cells in the TME (**Figure S1**B), promoting Tregs and macrophage infiltration, but further analysis also revealed a positive correlation between PTPN22 expression and Tumor Immune Dysfunction and Rejection (TIDE) immune dysfunction scores (**Figure 1**C).

Western blot analysis confirmed the upregulation of PTPN22 protein levels in all RCC cell lines when compared to primary normal HK2 cells (**Figure 1**D). The elevated expression of PTPN22 was further validated in paired RCC tissue samples using Immunohistochemistry (IHC) staining (**Figure 1**E, **S1**A). Accordingly, we detected PTPN22 and PD-L1 expression in parallel by patient-derived tissues using IHC (**Figure 1**F), and observed a positive correlation at the protein level (**Figure 1**G).

Although PTPN22 expression was not associated with patient prognosis in the TCGA-KIRC cohort, interestingly, in a cohort of metastatic ccRCC patients treated with ICI,^27^ high expression of PTPN22 was associated with improved overall survival, mirroring the response pattern observed with PD-L1 expression in ICI-treated patients (**Figure S1**C). Given this clinical parallel and correlative relationship, we hypothesized that PTPN22 directly regulates the expression of PD-L1.

To explore the regulatory relationship between PTPN22 and PD-L1, we knocked down PTPN22 in the 786-O and ACHN RCC cell lines by employing lentiviral shRNA. Interestingly, western blot and immunofluorescence (IF) analyses revealed a synchronous downregulation of PD-L1 (**Figure 1**H,J). Conversely, PTPN22 overexpression rescued PD-L1 downregulation due to PTPN22 knockdown (**Figure 1**I, K). Flow cytometry further confirmed the modulation of PD-L1 by PTPN22 (**Figure S2**A, B). These findings consistently suggest that PTPN22 correlates with PD-L1 levels, indicating that it functions as a mediator of immunosuppressive TME programming in RCC.

### 2.2 PTPN22 influences tumor growth by modulating T cell infiltration

To investigate the role of PTPN22 in tumor growth both *in vitro* and *in vivo*, we conducted Cell Counting Kit-8 (CCK8) assays to evaluate cell proliferation *in vitro* and performed subcutaneous homograft assays in nude mice *in vivo*. The results demonstrated that knockdown of PTPN22 did not directly impact the proliferation or growth of renal cancer cell lines either *in vitro* or *in vivo* (**Figure 1**L-M, **S2**C). Based on these observations, we hypothesized that PTPN22 may promote tumor progression by upregulating PD-L1 and fostering an immunosuppressive tumor microenvironment. To further test this hypothesis, we transplanted Renca cells with or without PTPN22 knockdown into the subcutaneous tissue of BALB/c mice. The results indicated that mice implanted with Renca cells in which PTPN22 was knocked down exhibited significantly slower tumor growth, as well as reduced final tumor size and weight, compared with the control group (**Figure 1**O-Q). To further assess T cell infiltration within tumor tissue, we conducted multiplex immunofluorescence staining for various T cell markers (**Figure S2**D). The results revealed that knockdown of PTPN22 led to a significant increase in infiltrating activated T cells, accompanied by a marked decrease in Treg cell infiltration within the tumor microenvironment (**Figure 1**R, S). Immunohistochemistry results showed that PD-L1 expression was significantly downregulated in tumor tissues following PTPN22 knockdown (**Figure S2**E, G). In summary, PTPN22 does not directly regulate the growth or proliferation of tumor cells; rather, it promotes tumor progression by modulating PD-L1 expression, which in turn alters T cell infiltration within the tumor immune microenvironment.

To determine whether PTPN22 modulates T cell infiltration and function through regulation of PD-L1 expression, we performed a PD-L1/PD-1 binding assay to visualize the interaction between PD-L1 and PD-1. Renal cancer cells subjected to various treatments were incubated with recombinant PD-1 protein and fluorescent antibodies, and the PD-L1/PD-1 interaction was subsequently detected by confocal microscopy. The intensity of green fluorescence reflected the degree of PD-1/PD-L1 interaction. Renal cancer cells with PTPN22 knockdown exhibited reduced green fluorescence, indicating that PTPN22 silencing diminishes the capacity of cancer cells to bind PD-1 by lowering PD-L1 levels; this effect was reversed by PD-L1 overexpression (**Figure S3**A, B).

To further assess the functional consequences of PD-L1 downregulation mediated by PTPN22 knockdown, we co-cultured renal cancer cells with Jurkat cells stably expressing both PD-1 and a NFAT luciferase reporter (**Figure S3**E). PD-1/PD-L1 binding results in T cell inactivation and loss of luminescent signal, whereas blockade of the PD-1/PD-L1 interaction reactivates NFAT signaling and restores luminescence. The results demonstrated that PTPN22 knockdown significantly increased bioluminescent signal, indicating disruption of PD-L1 checkpoint activity and enhanced T cell activation (**Figure S3**F). Conversely, PD-L1 overexpression reversed this effect, thereby promoting T cell exhaustion and inactivation (**Figure S3**F).

### 2.3 PTPN22 regulates PD-L1 expression by modulating ubiquitination

**Figure 2:**
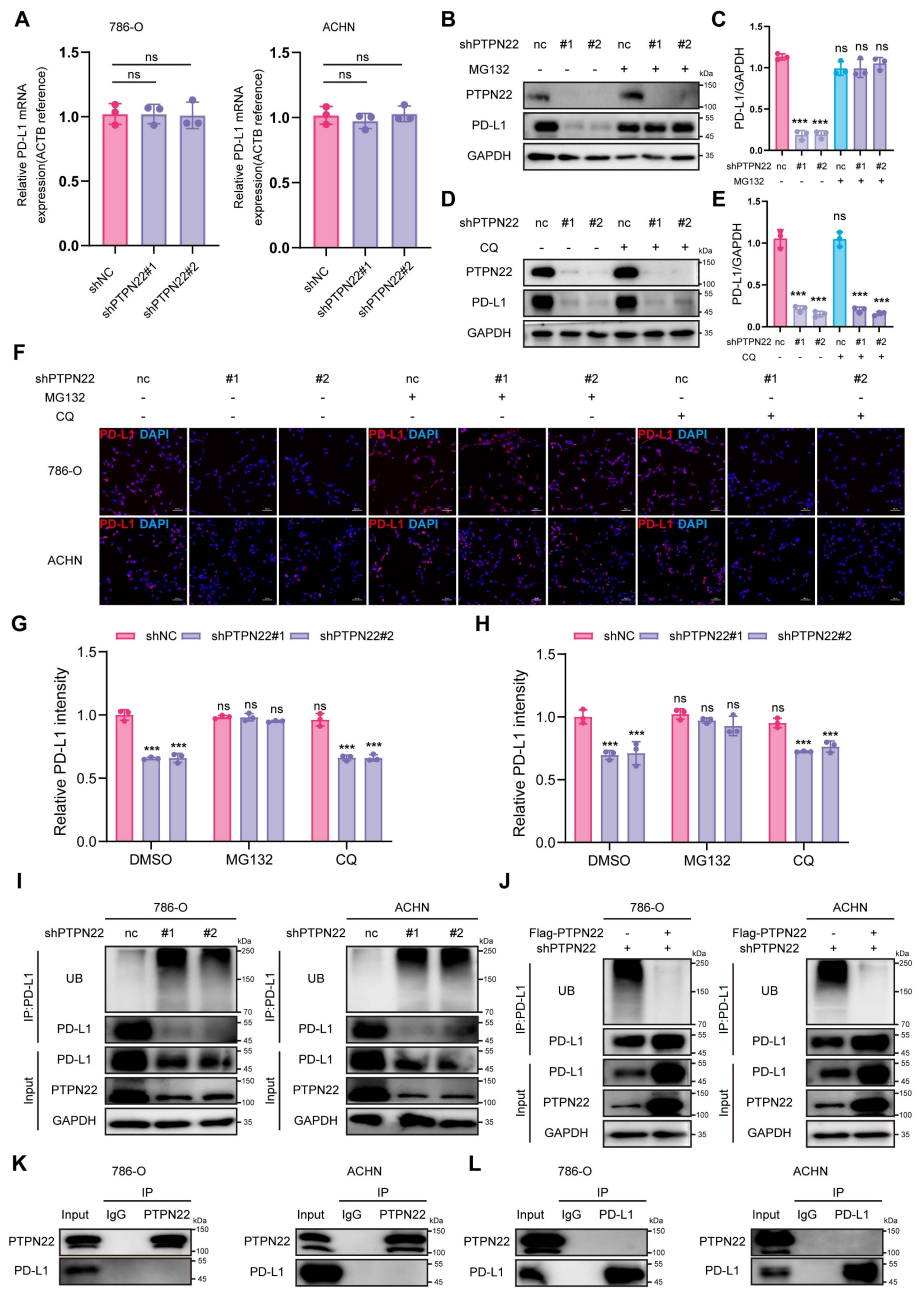
PTPN22 regulates PD-L1 expression by modulating ubiquitination. A) qRT-PCR analysis of PD-L1 mRNA in 786-O and ACHN cells after PTPN22 knockdown. B,C) Representative western blot and quantification of PTPN22 and PD-L1 protein levels in 786-O and ACHN cells after PTPN22 knockdown, with or without 10 μM MG132 for 24h. D,E) Representative western blot and quantification of PTPN22 and PD-L1 protein levels in 786-O and ACHN cells after PTPN22 knockdown, with or without 40 μM CQ for 24h. F) Representative IF images and quantification of PD-L1(red) in 786-O (G) and ACHN cells (H) after PTPN22 knockdown, treated with DMSO, 10 μM MG132, or 40 μM CQ for 24 h. Scale bar = 100 μm. I) Co-IP assays showing that increased PD-L1 ubiquitination in 786-O and ACHN cells after PTPN22 knockdown. J) Co-IP assays showing decreased PD-L1 ubiquitination in 786-O and ACHN cells after a rescue experiment following PTPN22 knockdown. K) Co-IP assays showing endogenous PTPN22 and PD-L1 interaction in 786-O and ACHN cells (IP: PTPN22). L) Co-IP showing endogenous PTPN22 and PD-L1 interaction in 786-O and ACHN cells (IP: PD-L1). ns, not significant; *p < 0.05, **p < 0.01 and ***p < 0.001.

To elucidate the mechanism of PTPN22-mediated PD-L1 regulation, we first detected PD-L1 mRNA levels following PTPN22 knockdown. qRT-PCR analysis revealed no significant change (**Figure 2**A), indicating an involvement of post-transcriptional regulation.

Since eukaryotic protein degradation primarily occurs through either the proteasome or lysosome pathways 28. we subsequently treated shPTPN22-expressing RCC cells with MG132 (a proteasome inhibitor) or CQ (a lysosome inhibitor). Western blot analysis showed MG132, but not CQ, rescued PD-L1 downregulation induced by PTPN22 knockdown (**Figure 2**B-E), indicating that PTPN22 regulates PD-L1 through the proteasome pathway. IF further demonstrated that the downregulation of PD-L1 on cell membranes could likewise be reversed by proteasomal inhibitors (**Figure 2**F-H).

Given that polyubiquitination targets substrates for proteasomal degradation 29, we proceeded to examine the ubiquitination status of PD-L1. PTPN22 knockdown significantly elevated PD-L1 poly-ubiquitination (**Figure 2**I), whereas subsequent PTPN22 overexpression reversed PD-L1 poly-ubiquitination (**Figure 2**J). Emerging evidence has shown that PD-L1 is subjected to phosphorylation at different serine (Ser), threonine (Thr), and tyrosine (Tyr) residues, which impact its protein stability and functions. Specifically, glycogen synthase kinase 3β (GSK3β) directly phosphorylates PD-L1 at the T180 and S184 sites, leading to its poly-ubiquitination by β-transducin repeat-containing protein (β-TRCP) 30. To determine whether PTPN22 directly binds to PD-L1 and alters its phosphorylation state to affect its ubiquitination levels, co-IP assays were performed. No direct interaction between PD-L1 and PTPN22 was observed (**Figure 2**K,L), suggesting that PTPN22 regulates PD-L1 stability indirectly through the ubiquitin-proteasome pathway.

### 2.4 PTPN22 associates with and dephosphorylates CBL at tyrosine 700

**Figure 3:**
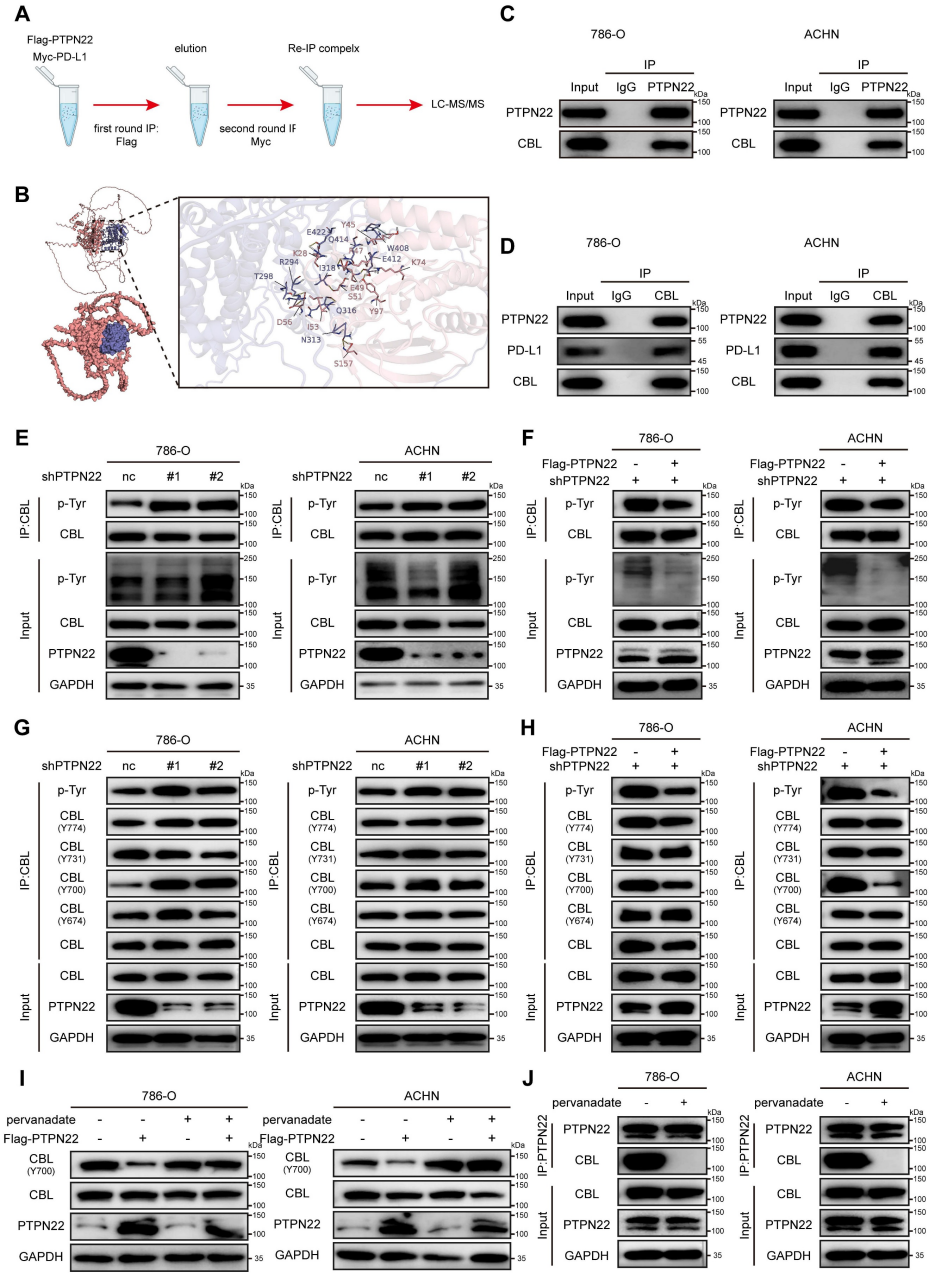
CBL is a substrate of PTPN22, which dephosphorylates it at tyrosine 700. A) Illustration indicated the two rounds IP in 786-O cells stably transfected with the indicated plasmids. B) Computer-performed molecular docking simulation of PTPN22 with CBL. (red: PTPN22, blue: CBL). C) Co-IP showing endogenous PTPN22 and CBL interaction in 786-O and ACHN cells (IP: PTPN22). D) Co-IP showing endogenous CBL, PTPN22 and PD-L1 interaction in 786-O and ACHN cells (IP: CBL). E) Co-IP showing increased CBL phosphorylation after PTPN22 knockdown in 786-O and ACHN cells. F) Co-IP showing decreased CBL phosphorylation in 786-O and ACHN cells after a rescue experiment following PTPN22 knockdown. G) Co-IP showing increased CBL Y700 phosphorylation after PTPN22 knockdown in 786-O and ACHN cells. H) Co-IP showing decreased CBL Y700 phosphorylation in 786-O and ACHN cells in 786-O and ACHN cells after a rescue experiment following PTPN22 knockdown. I) Representative western blot of PTPN22, CBL protein levels and CBL Y700 phosphorylation levels in 786-O and ACHN cells after PTPN22 overexpression, with or without 10 μM pervanadate (the tyrosine phosphatase inhibitor) for 15 min. J) Co-IP showing endogenous PTPN22 and CBL interaction in 786-O and ACHN cells (IP: PTPN22), with or without 10 μM pervanadate for 15 min.

To identify mediators of PTPN22-dependent PD-L1 degradation, we conducted a stepwise IP assay and LC-MS/MS to identify potential E3 ubiquitin ligase regulating PD-L1 ubiquitination and delineate how PTPN22-E3 ubiquitin ligase is coordinated (**Figure 3**A). Synthesizing the results of LC-MC/MC and the reported functions of protein, we prioritized CBL as the top candidate interacting with PTPN22 and regulating PD-L1 ubiquitination. As shown in **Figure 3**B, several stable hydrogen bonds formed between the backbone of PTPN22 and CBL. Structurally, CBL comprises several regions that encode various functionally distinct protein domains. The RING finger domain functions as an E3 ubiquitin ligase and mediates the ubiquitination of its targets. The C-terminus contains a proline-rich domain that serves as a protein-protein interaction domain, mediating interactions with diverse targets. Three exposed tyrosines in this region (Tyr700, Tyr731 and Tyr744) regulate interactions with downstream signaling molecules 31, 32. Since PTPN22 belongs to the PTP family that exert dephosphorylation 33, we hypothesized that PTPN22 may affect the E3 ubiquitin ligase activity of CBL by dephosphorylating specific tyrosine sites in CBL. Co-IP assays confirmed CBL's association with both PTPN22 and PD-L1 in 786-O and ACHN cells (**Figure 3**C,D).

To test the dephosphorylation function of PTPN22 on CBL, co-IP assays was performed. Results demonstrated that PTPN22 knockdown significantly increased tyrosine phosphorylation levels of CBL in RCC cells (**Figure 3**E), while subsequent PTPN22 overexpression significantly reversed phosphorylation levels (**Figure 3**F). To identify PTPN22's target site, we assessed the phosphorylation level at Y674, Y700, Y731, and Y774 of CBL. CBL Tyr700 was specifically dephosphorylated by PTPN22, as evidenced by elevated phosphorylation upon PTPN22 knockdown (**Figure 3**G) and reduced phosphorylation after PTPN22 overexpression (**Figure 3**H). Similarly, we analyzed subcutaneous tumor tissues derived from PTPN22-knockdown cells. Immunohistochemical analysis revealed a marked decrease in the protein level ofp-CBL Y700 in tumors with PTPN22 knockdown (**Figure S2**E, F). The sodium pervanadate (tyrosine phosphatase inhibitor) can also abolished Tyr700's dephosphorylation (**Figure 3**I), which is achieved by disrupting the binding between PTPN22 and CBL (**Figure 3**J). These findings collectively demonstrate that PTPN22 directly interacts with and dephosphorylates CBL at tyrosine 700.

### 2.5 CBL promotes K48-linked ubiquitination and degradation of PD-L1

**Figure 4:**
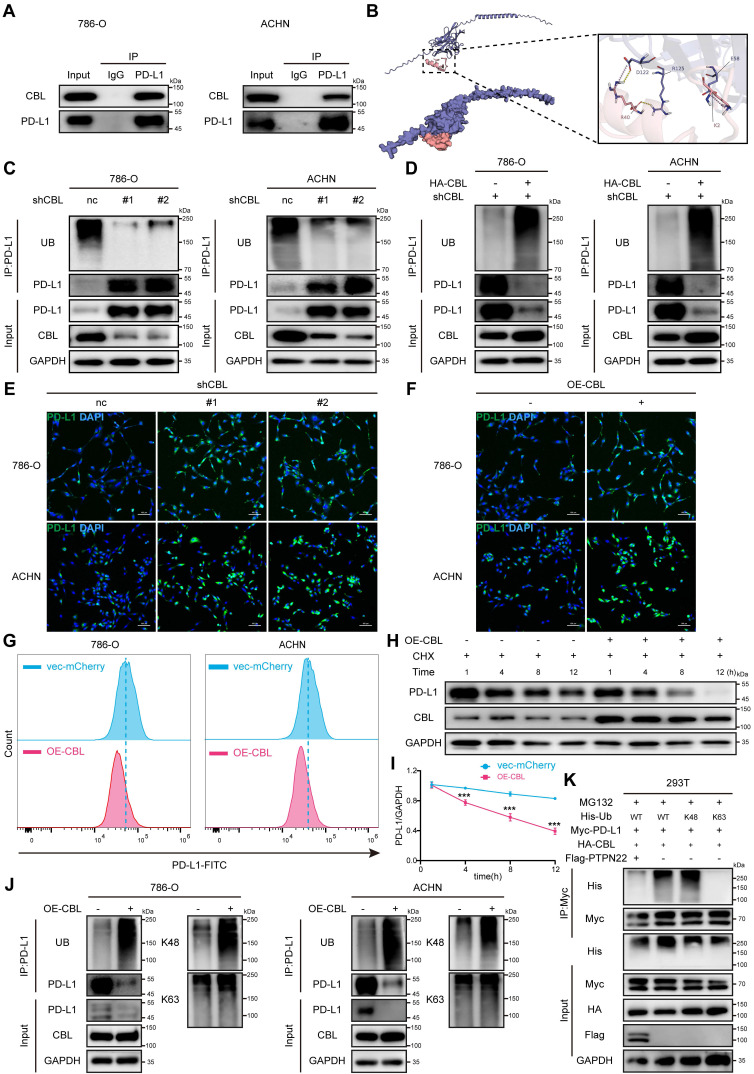
CBL binding promotes PD-L1 ubiquitination and reduces stability. A) Co-IP showing endogenous PD-L1 and CBL interaction in 786-O and ACHN cells (IP: PD-L1). B) Computer-performed molecular docking simulation of CBL with PD-L1 (red: CBL catalytic domain, blue: PD-L1). C) Co-IP showing decreased PD-L1 ubiquitination after CBL knockdown in 786-O and ACHN cells. D) Co-IP showing increased PD-L1 ubiquitination in 786-O and ACHN cells after a rescue experiment following CBL knockdown. E) Representative IF images of PD-L1 (green) in 786-O and ACHN cells after CBL knockdown. Scale bar = 100 μm. F) Representative IF images of PD-L1 (green) in 786-O and ACHN cells after CBL overexpression. Scale bar = 100 μm. G) Flow cytometry analysis of PD-L1 in 786-O and ACHN cells after CBL overexpression. H,I) Representative western blot and quantification from CHX assay showing PD-L1 protein stability after CBL overexpression in 786-O cells. Cells were treated with 50 ng/ml CHX for the indicated times. J) Co-IP showing increased PD-L1 K48-ubiquitination after CBL overexpression in 786-O and ACHN cells. K) Analysis of PD-L1 ubiquitin chain types catalyzed by CBL. 293T cells transfected with indicated constructs and treated with 10 μM MG132 for 24h. ns, not significant; *p < 0.05, **p < 0.01 and ***p < 0.001.

CBL is a RING finger E3 ubiquitin ligase that targets several proteins for degradation, such as receptor tyrosine kinases (c-Src, EGFR, PDGFR), non-receptor tyrosine kinases, mediators of Wnt signaling (β-catenin), and VEGF signaling (PLCγ1) 34-36. Co-IP assays confirmed the interaction between CBL and PD-L1 (**Figure 4**A), and molecular docking simulations revealed stable hydrogen bonds between CBL's catalytic domain and PD-L1 (**Figure 4**B).

To determine whether PD-L1 serves as a substrate for CBL, co-IP assays were performed. Results demonstrated that CBL knockdown significantly upregulated PD-L1 protein level and downregulated its poly-ubiquitination (**Figure 4**C), whereas CBL overexpression markedly decreased PD-L1 protein level and increased its poly-ubiquitination in RCC cells (**Figure 4**D). These finding were confirmed by IF and flow cytometry analyses (**Figure 4**E,F,G). CHX assays demonstrated that ectopic expression of CBL reduced the PD-L1 stability and accelerated its degradation (**Figure 4**H,I).

Previous studies have shown that CBL E3 ligase primarily facilitates the assembly of lysine (K) 48-linked poly-ubiquitin chain 37. To investigate the specific ubiquitin chain linkage involved in CBL-mediated PD-L1 ubiquitination, we used the anti-K48 and anti-K63 ubiquitin-specific antibodies to detect polyubiquitination by co-IP assays. It demonstrated that CBL overexpression caused robust K48-linked polyubiquitination of PD-L1, while K63-linked polyubiquitination remained unchanged (**Figure 4**J,K). Furthermore, we established a ubiquitination assay using Myc-PD-L1, HA-CBL, and His-tagged ubiquitin with only K63, or K48 (other lysines were mutated to arginines). Interestingly, coexpression of CBL caused K48 ubiquitin-dependent polyubiquitination of PD-L1, but not K63 ubiquitin-dependent polyubiquitination, and the ubiquitination was reversed by additional expression of PTPN22. These findings collectively demonstrate that CBL serves as an E3 ubiquitin ligase for PD-L1, promoting its degradation through K48-linked polyubiquitination.

In addition, we found that CBL overexpression effectively reduced the binding affinity between renal cancer cells and PD-1, an effect that was reversed by the overexpression of PD-L1 (**Figure S3**C, D). Similarly, CBL overexpression significantly increased the fluorescence intensity of NFAT luciferase in Jurkat cells, indicating that CBL overexpression inhibits the PD-L1/PD-1 interaction and promotes T cell activation (**Figure S3**G).

### 2.6 CBL Y700 phosphorylation level affects E3 ubiquitin ligase activity

**Figure 5:**
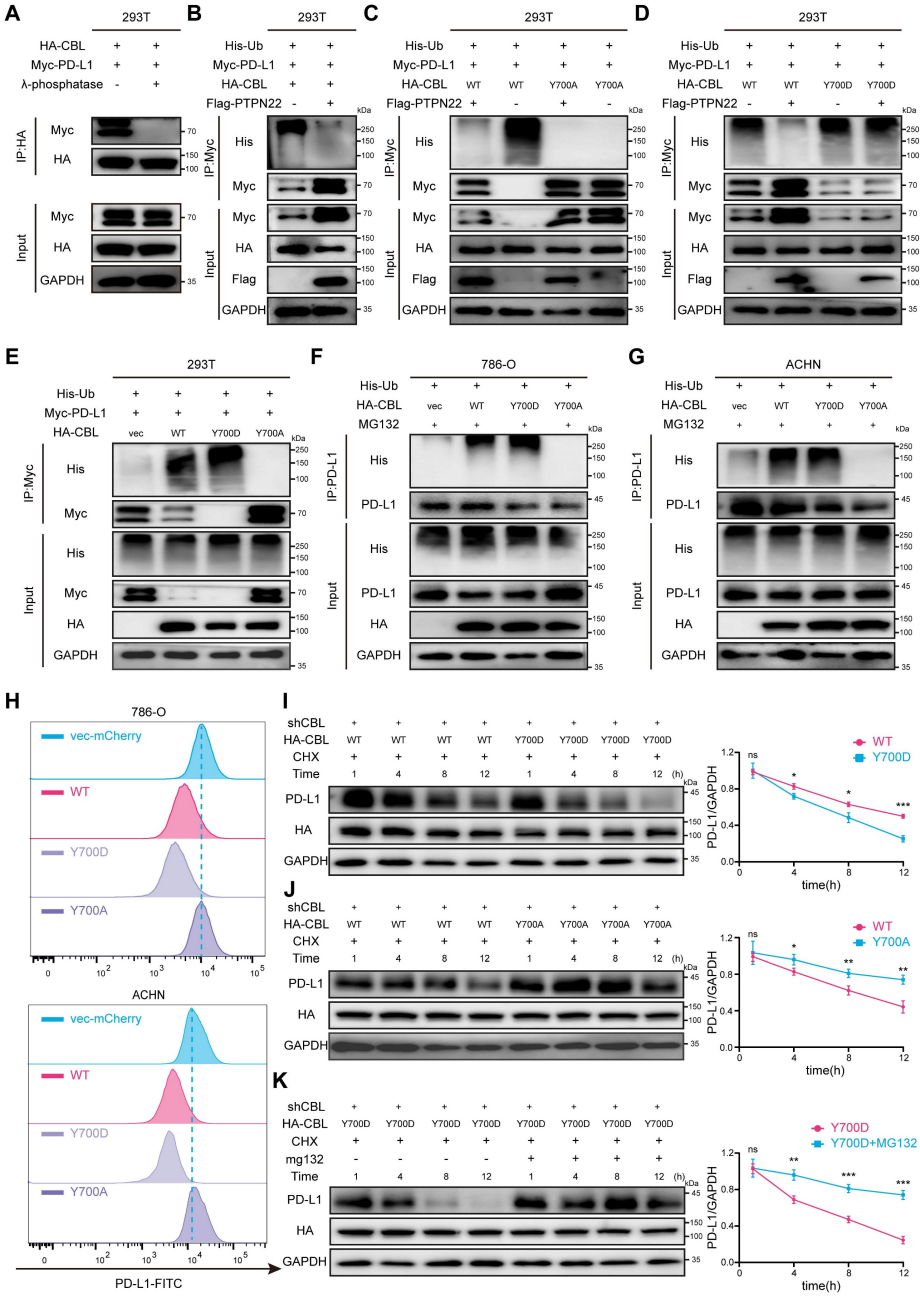
CBL Y700 phosphorylation level affects E3 ubiquitin ligase activity. A) Co-IP showing reduced CBL-PD-L1 interaction after λ-phosphatase treatment. 293T cell lysates were treated or not with 20 U/μl λ-phosphatase for 3 h at 30 °C. B) Co-IP showing decreased PD-L1 ubiquitination after PTPN22 overexpression in 293T cells. C) Co-IP showing loss of E3 ubiquitin ligase activity after CBL Y700 dephosphorylation in 293T cells. D) Co-IP showing enhanced E3 ubiquitin ligase activity after CBL Y700 phosphorylation in 293T cells. E) Co-IP showing CBL Y700 phosphorylation level affects E3 ubiquitin ligase activity in 293T cells. F,G) Co-IP showing CBL Y700 phosphorylation level affects E3 ubiquitin ligase activity in 786-O (F) and ACHN (G) cells. Cells were treated with 10 μM MG132 for 24h. H) Flow cytometry analysis of PD-L1 in 786-O and ACHN cells transfected with indicated constructs. I) Representative western blot (left panel) and quantification (right panel) from CHX assay showing PD-L1 protein stability in 786-O cells transfected with indicated constructs. Cells were treated with 50 ng/ml CHX for the indicated times. J) Representative western blot (left panel) and quantification (right panel) from CHX assay showing PD-L1 protein stability in 786-O cells transfected with indicated constructs. Cells were treated with 50 ng/ml CHX for the indicated times. K) Representative western blot (top panel) and quantification (bottom panel) from CHX assay showing PD-L1 protein stability in 786-O cells transfected with indicated constructs. Cells were treated with or without 10 μM MG132 for 24h, 50 ng/ml CHX for the indicated times. ns, not significant; *p < 0.05, **p < 0.01 and ***p < 0.001.

To investigate the impact of CBL phosphorylation on its interaction with PD-L1, we conducted *in vitro* dephosphorylation assays and co-IP assays. Co-transfection of HA-CBL and Myc-PD-L1 in 293T cells revealed that dephosphorylated CBL failed to bind PD-L1 (**Figure 5**A). Subsequently, we introduced His-Ub and Flag-PTPN22 into 293T cells. Co-IP assay revealed that PTPN22 overexpression reduces CBL-mediated ubiquitination of PD-L1 (**Figure 5**B).

To specifically clarify the effect of CBL tyr700 phosphorylation on its E3 ligase activity, we constructed 700T→A (Y700A) and 700T→D (Y700D) mutants to simulate dephosphorylation and phosphorylation states, respectively. Co-IP assays demonstrated that CBL Y700A failed to ubiquitinate and degrade PD-L1 compared to CBL wild type (wt), and that PTPN22 overexpression reversed CBL wt-mediated PD-L1 ubiquitination (**Figure 5**C). Conversely, co-IP assays revealed that CBL Y700D exhibited enhanced E3 ubiquitin ligase activity, resulting in more pronounced PD-L1 ubiquitination and degradation compared to CBL wild type (**Figure 5**D). To validate the existence of the PTPN22/p-CBL Y700/PD-L1 axis, we performed IHC analysis on patient-derived tissue samples. Quantitative analysis revealed that the expression level of p-CBL Y700 was negatively correlated with both PTPN22 and PD-L1, and these correlations were statistically significant (**Figure S4**A, B, C).

To compare CBL's E3 ubiquitin ligase activity against exogenous PD-L1, we transfected 293T with vector, CBL wt, CBL Y700A, and CBL Y700D, respectively. Co-IP assays demonstrated that CBL possessed ubiquitination activity to degrade PD-L1, which was abolished in Y700A and enhanced in Y700D mutant (**Figure 5**E). To further investigate CBL's E3 ubiquitin ligase activity against endogenous PD-L1, we transfected 786-O and ACHN cells with vector, CBL wt, CBL Y700A, and CBL Y700D, respectively.

Co-IP assays corroborated these findings (**Figure 5**F,G). Flow cytometry analysis revealed that CBL wt significantly reduced cell surface PD-L1, and CBL Y700D showed pronounced downregulation compared to CBL wt, while CBL Y700A had no effect on PD-L1 expression in 786-O and ACHN cells (**Figure 5**H).

CHX assays were performed to examine the effects of CBL mutations on PD-L1 degradation. Compared to CBL wt, CBL Y700D exhibited a faster PD-L1 degradation rate, while CBL Y700A showed a slower PD-L1 degradation rate. The degradation of PD-L1 by CBL Y700D was reversed by MG132 treatment (**Figure 5**I,J,K).

Collectively, our finding indicates that tyrosine 700 phosphorylation of CBL regulates its ubiquitin ligase activity towards PD-L1. In its phosphorylated state (low PTPN22 expression), CBL binds to PD-L1 and promotes its degradation via the ubiquitin-proteasome pathway. Conversely, in its in dephosphorylated state (high PTPN22 expression), CBL fail to interact with PD-L1, leading to its elevated expression.

### 2.7 Curcumin inhibits PTPN22 activity through direct binding to PTPN22

**Figure 6:**
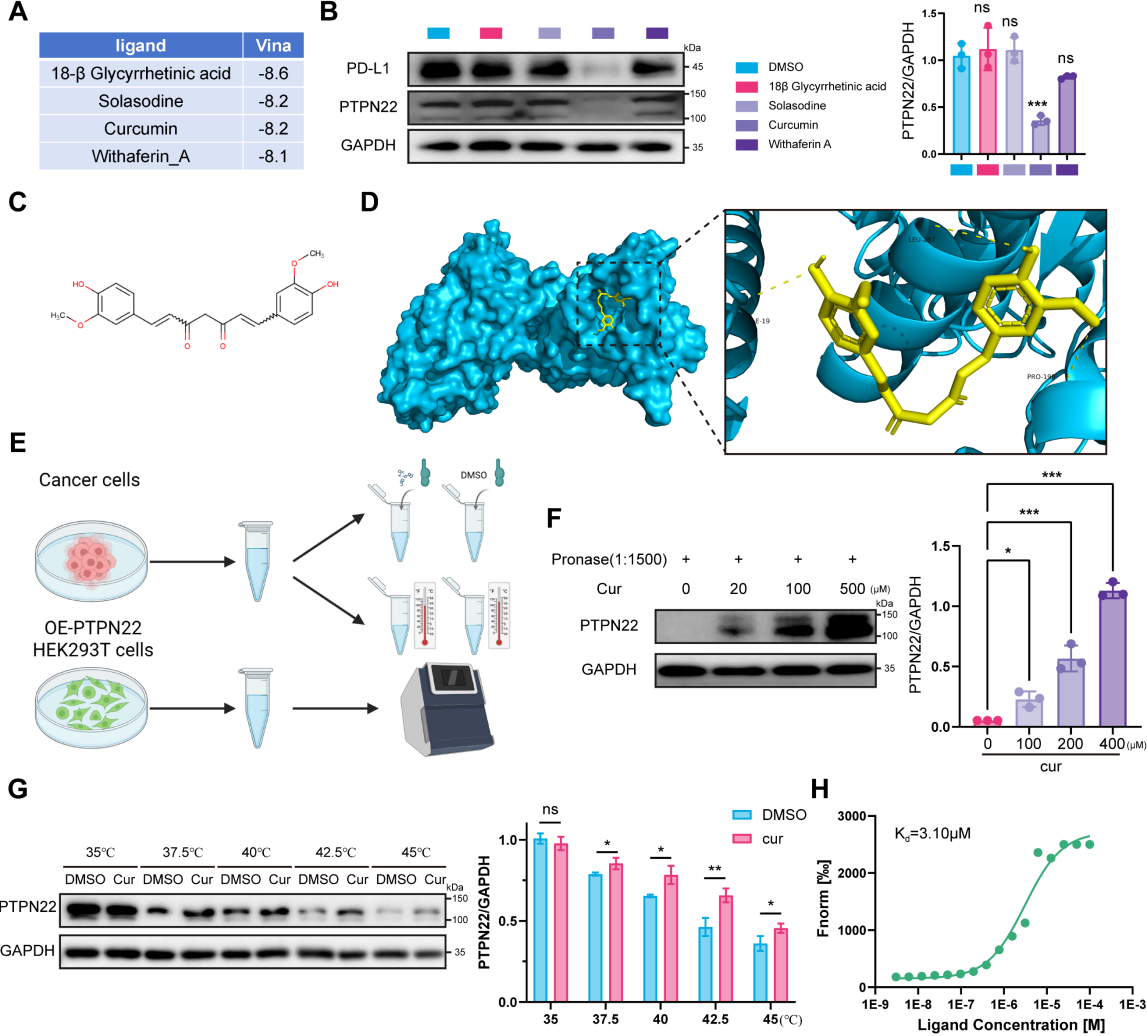
Curcumin binds PTPN22 and is a potential inhibitor of PTPN22. A) Top 4 monomers from molecular docking of 114 traditional Chinese medicine monomers with PTPN22. B) Representative western blot (left panel) and quantification (right panel) showing that curcumin effectively downregulates PTPN22 and PD-L1 in 786-O cells. Cells were treated with 100μM 18β-Glycyrrhetinic Acid, 10 μM Solasodine, 20 μM Curcumin or 4 μM Withaferin A for 72 h. C) Structural formula of curcumin. D) Computer-performed molecular docking simulation of curcumin with PTPN22 (blue: PTPN22, yellow: curcumin). E) Flowchart for DARTS, CETSA and MST assays. 786-O lysates for DARTS/CETSA, 293T PTPN22-GFP lysates for MST. F) Representative western blot (left panel) and quantification (right panel) showing curcumin's effect on PTPN22 stability at various concentrations when the ratio of pronase to protein was 1:1500. G) Representative western blot (left panel) and quantification (right panel) showing curcumin's effect on PTPN22 thermal stabilization at temperatures from 35 °C to 45 °C. H) The cellular MST assay of 293T cells with GFP-tagged PTPN22. ns, not significant; *p < 0.05, **p < 0.01 and ***p < 0.001.

In recent years, small molecule antagonists targeting intracellular negative regulators of immune responses have emerged as a new class of therapeutic agents for cancer immunotherapy. These inhibitors aim to enhance anti-tumor immune response and prolong the efficacy of checkpoint inhibition 38. To identify PTPN22 inhibitors, we leveraged traditional Chinese medicine monomers, a rich source of bioactive compounds, screening for antitumor agents with anti-inflammatory properties in response to the rapid advancement of RCC immunotherapy. From 1616 traditional Chinese medicine monomers, 114 compounds (Table S4, Supporting Information) were selected based on their documented relevance to tumor and inflammation according to the literature 39. These compounds, along with PTPN22, were processed using MOE software for molecular docking studies using the induced fit mode. Ultimately, four compounds (18β Glycyrrhetinic acid, Solasodine, Curcumin, Withaferin A) were identified as potential PTPN22 inhibitors (**Figure 6**A, Table S5). Western blot analysis demonstrated significant downregulation of PTPN22 protein in 786-O cell line after treatment with curcumin (**Figure 6**B).

Curcumin is the primary bioactive component of turmeric extracts (**Figure 6**C). As a natural plant monomer, it has gained significant attention as a dietary supplement due to its diverse pharmacological activities, including anti-inflammation, antioxidation, antitumor, and immune-modulatory properties 40-42. To validate our hypothesis, molecular docking were conducted to investigate the binding interaction between PTPN22 and curcumin. As shown in **Figure 6**D, three stable hydrogen bonds were observed between the backbone of Ile19, Leu287, Pro199, and curcumin.

To demonstrate direct binding between curcumin and PTPN22, we conducted a series of validation experiments (**Figure 6**E). Drug affinity responsive target (DARTS) and cellular thermal shift assay (CETSA) were employed to investigate direct binding between PTPN22 and curcumin, based on the biophysical principle that target protein exhibits thermal stabilization and resistance to degradation upon ligand binding 43, 44. DARTS and CETSA assays confirmed that co-incubation of PTPN22 with curcumin enhanced its thermal stability and resistance to degradation, indicating direct binding between the two molecules (**Figure 6**F,G). We further investigated the direct binding between curcumin and PTPN22 using microscale thermophoresis (MST) with exogenous PTPN2-GFP fusion protein. As expected, curcumin bound to PTPN22 with a K_d_ value of 3.10 μM (**Figure 6**H). Collectively, these results demonstrate that curcumin binds to and decreases the levels of PTPN22.

### 2.8 Curcumin degrades PTPN22 via the ubiquitin-proteasome pathway and improves tumor immune microenvironment

**Figure 7:**
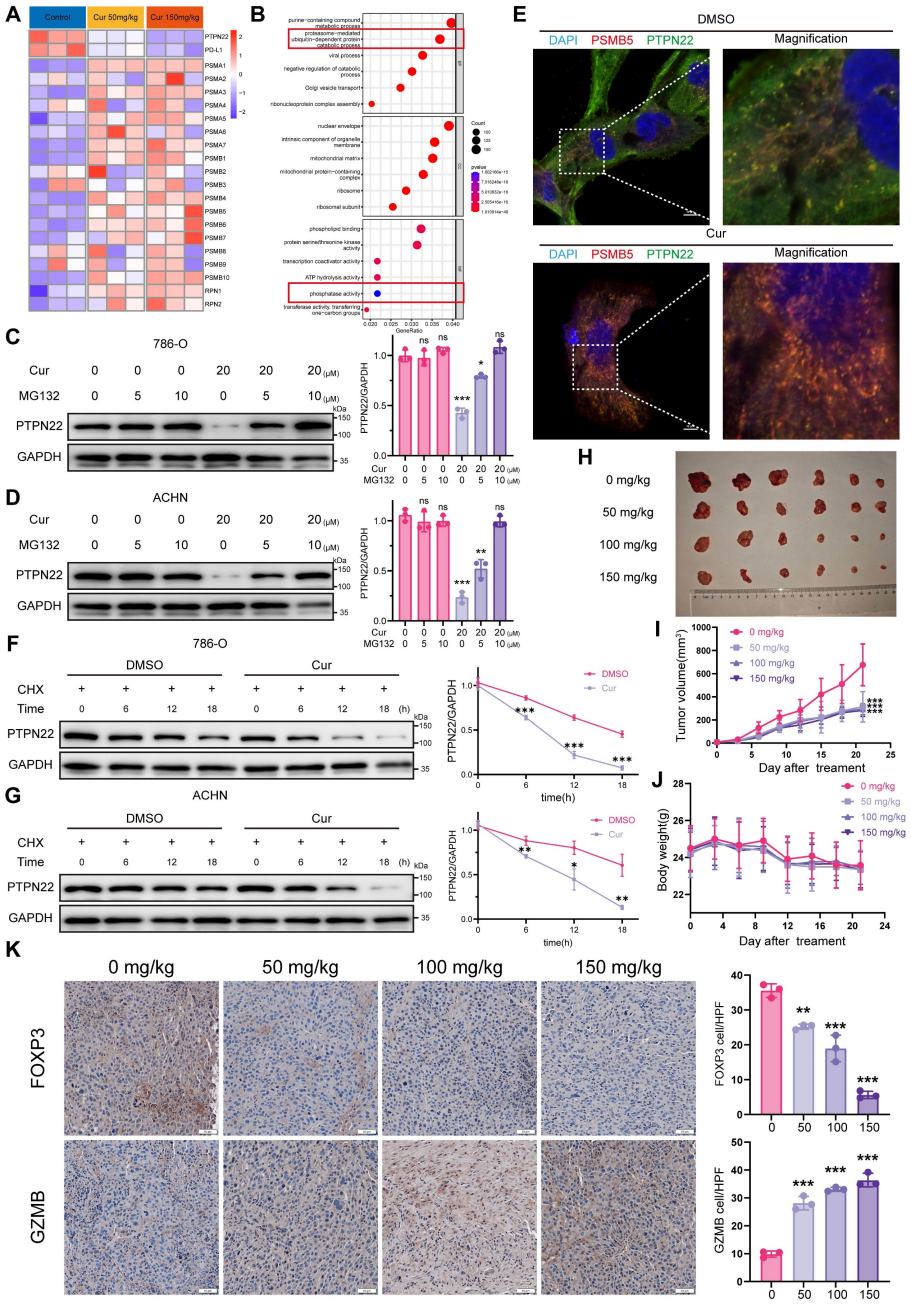
Curcumin decreases PTPN22 stability and improves tumor immune microenvironment. A) Heatmap showing the protein expression of mouse-derived tumors treated with curcumin. B) GO analysis of differentially expressed proteins in mouse-derived tumors. C) Representative western blot (left panel) and quantification (right panel) showing PTPN22 protein levels in 786-O cells treated with 20 μM curcumin. Cells were treated with 0, 5, or 10 μM MG132 for 24 h. D) Representative western blot (left panel) and quantification (right panel) showing PTPN22 protein levels in ACHN cells treated with 20 μM curcumin. Cells were treated with 0, 5, or 10 μM MG132 for 24 h. E) Representative IF images of nucleus (blue), PTPN22 (green) and PSMB5 (red) in 786-O cells treated with 20 μM curcumin. Scale bar = 10 μm. F) Representative western blot (left panel) and quantification (right panel) from CHX assay showing PTPN22 protein stability in 786-O cells treated with 20 μM curcumin. Cells were treated with 50 ng/ml CHX for the indicated times. G) Representative western blot (left panel) and quantification (right panel) from CHX assay showing PTPN22 protein stability in ACHN cells treated with 20 μM curcumin. Cells were treated with 50 ng/ml CHX for the indicated times. H,I,J) Representative images (H), growth curves (I) and body weight curves (J) of BALB/c mice with indicated treatment. (n = 9 per group) K) Representative IHC staining images (left panel) and quantification (right panel) for GZMB and FOXP3 in BALB/c mice with indicated treatment. ns, not significant; *p < 0.05, **p < 0.01 and ***p < 0.001.

To elucidate the molecular mechanism underlying PTPN22 degradation by curcumin, we conducted proteomic analysis on tumors from curcumin-treated mice. Compared to controls, 55 differentially expressed proteins (DEPs) (pvalue < 0.05, fold change > 1) were identified in the low-dose group (50 mg/kg) and 71 DEPs in the high-dose group (150 mg/kg) (**Figure S5**A,B). To assess the reproducibility of our findings, we utilized principal-component analysis (PCA) to visualize the variation among different protein types. Analysis of the PCA graph and heatmap revealed a high degree of homogeneity among repeats, confirming the reliability of our data (**Figure S5**C,D). Notably, several proteasome-associated proteins, including PSMB5, PSMB6, PSMB7 and PSMB10, were upregulated, supporting the conclusion that curcumin modulates proteasome function (**Figure 7**A). Additionally, both PD-L1 and PTPN22 were observed to be downregulated (**Figure 7**A). GO analysis revealed significant upregulation of pathways associated with proteasome-mediated ubiquitin-dependent protein catabolic process and phosphatase activity (**Figure 7**B).

CCK8 assay indicated that curcumin effectively inhibit tumor cell growth (**Figure S6**A). Moreover, curcumin treatment significantly reduced PTPN22 protein in 786-O and ACHN cells in time- and concentration-dependent manners, without affecting mRNA levels (**Figure S6**C-D). Given apparent activation of the ubiquitin-proteasome pathway, we co-treated cells with curcumin and MG132, which reversed the curcumin-mediated inhibition of PTPN22 (**Figure 7**C,D).

To investigate the impact of curcumin on PTPN22 within the ubiquitin-proteasome pathway, we examined the interaction between PTPN22 and the proteasome. As anticipated, curcumin treatment significantly increased the co-localization of PTPN22 with the PSMB5-tagged proteasome (**Figure 7**E). Furthermore, In the presence of CHX, the turnover rate of PD-L1 in curcumin-treated cells was significantly faster than in untreated cells (**Figure 7**F,G). Taken together, these findings suggest that curcumin-induced degradation of PTPN22 occurs through a proteasome-dependent mechanism.

To evaluate the potential antitumor activity of curcumin *in vivo*, we administered either solvent control or curcumin to mice bearing Renca tumors once daily for 21 days. Curcumin treatment resulted in significant inhibition of Renca tumor growth in mice, with observed inhibition rates of 46.4%, 45.5% and 39.6% at dosages of 50, 100 and 150 mg/kg, respectively (**Figure 7**H,I). Additionally, curcumin treatment significantly reduced the tumor weight in comparison to the controls (**Figure S7**A). There was no significant difference in body weight among the various groups of mice, and H&E staining revealed no apparent toxicity in the liver, spleen or kidney (**Figure 7**J, **S7**B,C,D,E). To validate that curcumin downregulates PD-L1 expression via the PTPN22/p-CBL Y700/PD-L1 axis, we evaluated the expression levels of PTPN22, p-CBL Y700, and PD-L1 in tissue samples (**Figure S8**A). Consistent with our previous findings, the expression levels of PTPN22 and PD-L1 were downregulated in a dose-dependent manner, whereas p-CBL Y700 expression was dose-dependently upregulated (**Figure S8**B, C, D). To evaluate whether curcumin enhances T cell activation and cytotoxic capacity while improving the tumor microenvironment, we performed PD-L1/PD-1 blockade assay and analyzed factors associated with T cell activation and cytotoxicity (FOXP3, GZMB, c-caspase-3 and IFN-γ). Curcumin significantly induced transcription-mediated bioluminescent signaling in a dose-dependent manner, indicating that curcumin promotes T cell activation by downregulating PD-L1 expression (**Figure S3**H). Results indicated that the T cell activation markers GZMB and IFN-γ were significantly increased in the tumor, while the immunosuppressive factor FOXP3 was significantly reduced following curcumin treatment (**Figure 7**K, **S8**E, F, G). This suggests that curcumin effectively downregulates PTPN22/p-CBL Y700/PD-L1 and promotes T cell activation, thereby enhancing the tumor immune microenvironment and inhibiting renal cancer growth.

### 2.9 Curcumin synergizes with immune checkpoint inhibitors to enhance antitumor efficacy

**Figure 8:**
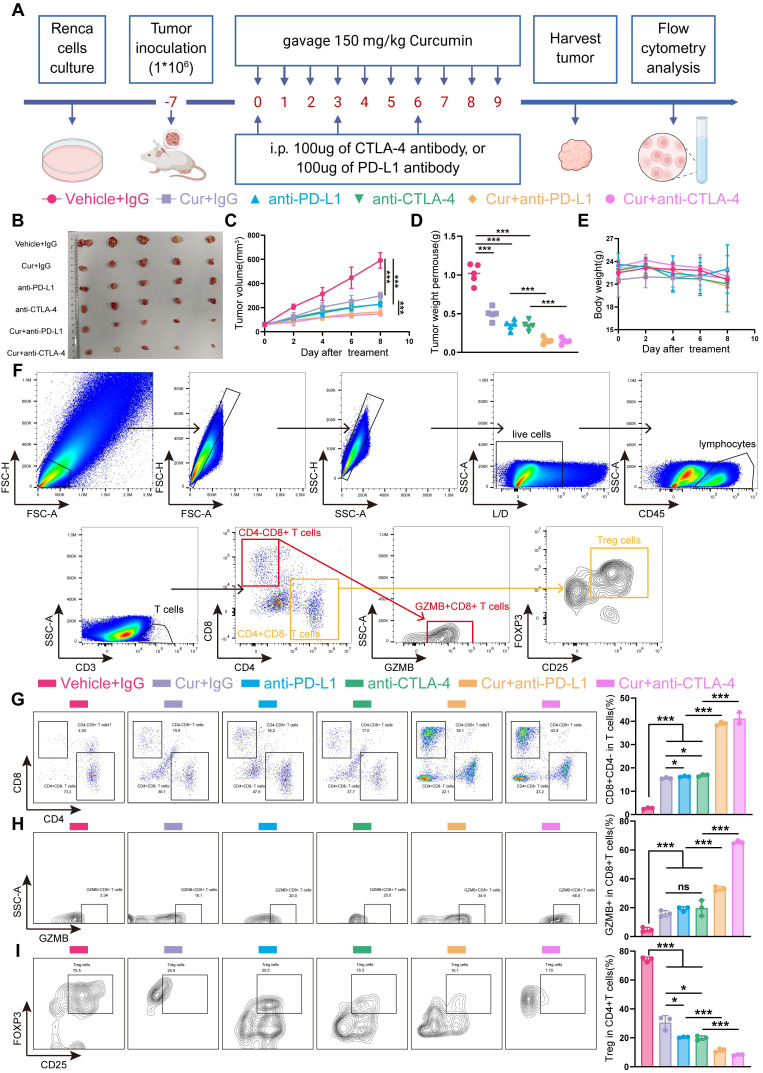
Curcumin enhances ICI efficacy and inhibits tumor growth. A) Treatment schedule: Renca cells injected into BALB/c mice on day -7, and curcumin, anti-PD-L1, and anti-CTLA4 were administered as indicated. B,C,D,E) Representative images (B), growth curves (C), tumor weight (D) and body weight curves (E) of BALB/c mice with indicated treatment. (n = 5 per group). F) Flow cytometry gating strategy for quantify CD4-CD8+ T cells, CD4+CD8- T cells, CD4-CD8+GZMB+ T cells (activated CD8+ T cells) and CD4+CD8-CD25+FXOP3+ (Tregs). G) Representative flow cytometry images (left panel) and quantification (right panel) of CD4+CD8- T cells and CD4-CD8+ T cells from tumors with indicated treatments. H) Representative flow cytometry images (left panel) and quantification (right panel) of CD4-CD8+GZMB+ T cells from tumors with indicated treatments. I) Representative flow cytometry images (left panel) and quantification (right panel) of Tregs from tumors with indicated treatments. ns, not significant; *p < 0.05, **p < 0.01 and ***p < 0.001.

Clinical evidences demonstrate that anti-PD-1/anti-CTLA4 combination therapy outperforms PD-1 monotherapy, significantly improving both response rate and survival outcomes in cancer patients 42, 45. We therefore investigated the potential synery between curcumin and anti-CTLA4 or anti-PD-L1 immunotherapy. In murine models (**Figure 8**A), while curcumin, anti-PD-L1, or anti-CTLA4 monotherapy significantly reduced tumor growth rates, combination treatments further decreased tumor growth rates, volumes, and weights (**Figure 8**B-D). Critically, compared to single-agent ICIs, curcumin-ICIs combinations did not result in additional immune-related adverse events (irAEs) (**Figure 8**E, **S9**A,B,C,D).

To further explore the underlying mechanisms, we evaluated treatment effects on tumor-infiltrating lymphocytes (TILs), including CD4-CD8+ T cells, CD4+CD8- T cells, CD4-CD8+GZMB+ T cells (activated CD8+ T cells) and CD4+CD8-CD25+FXOP3+ (Tregs) (**Figure 8**F). Analysis of TILs revealed that curcumin improved the CD4-CD8+ T cell ratio, though to a lesser extent than the monotherapy group, while the enhancement of CD8+GZMB+ T cells was comparable to that observed in the monotherapy group. However, we observed that both the percentage of CD4-CD8+ T cells and CD8+GZMB+ T cells were significantly higher in the combination treatment groups compared to monotherapy alone (**Figure 8**G,H). Since GZMB levels in TILs serves as a marker of cytotoxic T cell activity 46, these results support the enhanced efficacy of curcumin combined with anti-CTLA4 or anti-PD-L1.

Tregs represent a major immunosuppressive populations in the TME that can facilitate tumor immune evasion by suppressing T lymphocyte immunity 47. Flow cytometry analysis showed a significant reduction in Tregs accumulation in the combination treatment groups compared to single-agent treatments (**Figure 8**I).

Collectively, these results indicate that curcumin synergizes with anti-CTLA4 or anti-PD-L1 therapy exhibits synergistic antitumor effect. Our findings suggest that curcumin represents a promising adjuvant therapy to enhance antitumor immunity and improve the efficacy of anti-CTLA4 or anti-PD-L1 immune checkpoints inhibitors.

## 3. Discussion

TILs have been extensively documented to confer survival benefits across various cancer types, including lung, colon, and breast cancers 48. Elevated CD8+ T cells infiltration within the tumor microenvironment is associated with favorable prognostic indicators and can predict response to ICI in specific cancer types 49, 50.

RCC presents a paradoxical immunological landscape: despite abundant TILs, RCC patients exhibit limited clinical benefit from endogenous or therapy-induced immune responses 5, 51. This disconnects stems from profound CD8+ T-cell exhaustion, characterized by elevated PD-1/CTLA-4 expression and dysfunctional cytotoxicity 52, 53.

PD-L1, a type I integral membrane glycoprotein expressed in various tumor types,^54^ plays a crucial role in immune evasion. Under normal conditions, activated antigen-specific cytotoxic T lymphocytes can recognize and mount direct cytotoxic responses against tumor cells 55. However, tumor cells overexpressing PD-L1 exhibit enhanced viability. When PD-L1 binds to PD-1, it leads to exhaustion of CD8+ cytotoxic T cells within the tumor, impairs tumor-reactive cytolytic T cell responses, enhances Tregs suppression, and broadly suppresses immune activation. This coordinated immunosuppression facilitates immune evasion and enables tumor cells to invade adjacent tissues 55. ICIs can restore sufficient numbers of functional tumor-specific T cells to eliminate cancer cells 56. While ICIs effectively reverses T cell exhaustion in the immune microenvironment of renal cancer, most renal cancer patients initially respond well but develop resistance in advanced stages 4. This highlights the need for further investigation into the molecular mechanisms underlying immune suppression and immune escape within the RCC microenvironment.

Recent research has primarily focused on PTPN22's role in autoimmune diseases and immune cells, with limited understanding of its function in tumor cells 23-25. Our study elucidates a previously unrecognized mechanistic axis driving this immunosuppression, centered on PTPN22-mediated stabilization of PD-L1 via phosphoregulation of the E3 ligase CBL. We establish PTPN22 as significantly overexpressed in RCC tumors, correlating with both immune dysfunction and PD-L1 protein levels. Functional studies reveal that PTPN22 post-translationally regulates PD-L1 stability without affecting its transcription, operating through the ubiquitin-proteasome pathway. Mechanistically, PTPN22 directly dephosphorylates CBL at Tyr700—a key allosteric site controlling its E3 ubiquitin ligase activity 31, 34. This dephosphorylation inactivates CBL, preventing K48-linked polyubiquitination and subsequent proteasomal degradation of PD-L1 21. Using phospho-defective (Y700A) and phosphomimetic (Y700D) mutants, we demonstrate that Tyr700 phosphorylation status dictates CBL's capacity to target PD-L1 for destruction, thereby controlling immunosuppressive PD-L1 abundance in the tumor microenvironment.

Compared to synthetic compounds, herbal monomers offer valuable resources for discovering novel anticancer drugs with higher biological activity and diverse structures 57. In this study, curcumin was selected as a drug candidate through molecular docking and experimental validation. Curcumin has been reported to inhibit various tumors, including non-small cell lung cancer, breast cancer, and hepatocellular carcinoma, while improving the immune microenvironment 40, 41. Our study demonstrated that curcumin directly binds to PTPN22, reducing its stability and decreasing PD-L1 expression. Animal experiments showed that curcumin activates T cells in the tumor microenvironment. Additionally, curcumin not only reduced PD-L1 levels in tumor cells but also promoted CD8+ T cell infiltration and activation into GZMB+ T cells while reducing Treg infiltration. Our results suggest that curcumin attenuates Treg accumulation and promotes T cell activation by inhibiting PTPN22-dependent PD-1/PD-L1 interaction. The development of ICI began with targeting two immunosuppressive molecular pathways: PD-1/PD-L1 and CTLA4/B7-1/2, resulting in significant clinical therapeutic efficacy. Since curcumin blocks the PD-L1/PD-1 pathway, combination therapy with curcumin and anti-CTLA4 may enhance tumor immunity. Furthermore, the combination of curcumin with PD-L1 blockers or CTLA-4 blockers may have a synergistic effect compared to monotherapy, further inhibiting tumor growth, promoting T-cell activation, inhibiting Treg infiltration, and dramatically improving TME. Importantly, curcumin does not cause significant toxicity compared to the irAEs associated with antibody-based ICIs. Despite limitations such as poor water solubility, low bioavailability, and high *in vivo* doses, curcumin showed no significant organ toxicity and has potential for development of curcumin related preparation.

These findings position PTPN22 as a theraputically tractable immune checkpoint regulating PD-L1 stability through phospho-switch control of CBL. The PTPN22-CBL-PD-L1 axis represents a clinically targetable resistance mechanism in RCC, with dual implications: (1) PTPN22 expression may serve as a biomarker for ICI response stratification, and (2) pharmacological inhibition of this pathway (e.g., via curcumin derivatives) could overcome resistance to existing immunotherapies. While curcumin's pharmacokinetic limitations warrant further optimization, our work provides a mechanistic foundation for next-generation combination therapies that simultaneously target canonical immune checkpoints and their upstream stabilizers.

## 4. Materials and Methods

### 4.1 Tissue samples and cell culture

The samples of clear cell renal cell carcinoma (ccRCC) and corresponding non-tumor kidney tissues were collected from hospitalized patients at the First Affiliated Hospital of Zhejiang Chinese Medical University (Zhejiang Provincial Hospital of Chinese Medicine). Prior to tissue collection and usage, written informed consent was obtained from all participants. Approval for this study was granted by the Research Ethics Committee of the First Affiliated Hospital of Zhejiang Chinese Medical University (approval no.2023-KLS-181-01, date of approval: April 26, 2024). Sample collection was conducted in accordance with the 1975 Declaration of Helsinki guidelines. Detailed clinicopathological data of these samples are provided in Table S1 (Supporting Information). All cell lines used in the study were acquired from the Cell Bank of the Chinese Academy of Sciences (Shanghai, China). HEK-293T cells and ACHN cells were cultured in Duchenne's Modified Eagle Medium (DMEM), and 786-O cells and Renca cells were grown in Roswell Park Memorial Institute (RPMI)-1640 medium. Both media were supplemented with 10% Fetal Bovine Serum (FBS, Gibco, USA) and penicillin-streptomycin and maintained at 37 ºC in a humidified incubator under 5% CO2.

### 4.2 Plasmid construction, transfection, and lentiviral packaging

The shRNAs of PTPN22 and CBL were synthesized by TranSheepBio (Shanghai, China) and subsequently cloned into the pLKO.1 vector. A detailed summary of the primers employed in this study can be found in Table S2 (Supporting Information). The full-length or mutant CBL, PTPN22 and PD-L1 genes were amplified via PCR and cloned into phage-Flag, phage-Myc or phage-HA vectors to produce PTPN22-Flag, CBL-wt-HA, PD-L1-Myc, CBL-Y700D-HA and CBL-Y700A-HA constructs. The plasmids were designed by Vigenebio (Jinan, China) and transfected into HEK-293T, 786-O, and ACHN cells using Lipo3000 (Thermo Fisher, NY, USA). Cells were co-transfected with the target plasmids and lentiviral packaging/envelope plasmids (psPAX2 and pMD2.G). After 12 hours of transfection, the medium was replaced with fresh media. Viral supernatants were harvested 72 h post-transfection and co-incubated with target cells in the presence of polybrene (Yeasen, Shanghai, China) for 8 h, after which the medium was again replaced with fresh media. Subsequently, the infected cells were cultured in the medium containing 2 μg/ml puromycin (Solarbio, China) or 800 ug/ml G418 (Solarbio, China) for a minimum of one week.

### 4.3 Animal models

All animal procedures were approved by the Animal Research Ethics Committee of the Hangzhou Institute of Medicine, Chinese Academy of Sciences (approval number AP2024-12-0390), and were conducted in strict accordance with the institution's ethical guidelines. Four-week-old male BALB/c mice were bred and maintained under specific pathogen-free (SPF) conditions. According to the experimental design, each mouse was injected with 1*10^6^ cells into the right axilla. The mice were then randomly divided into groups (nine mice per group) using a random number table and were gavaged with curcumin at dose of 0, 50, 100 and 150 mg/kg (Solarbio, China). Tumor growth was monitored every three days, and mouse weight was recorded. Tumor volume was quantified by measuring the length and width of the xenograft tumors and calculating the volume using the formula: (length*width^2^) / 2. All mice were sacrificed when the most giant grafted tumor reached a volume of 1000 mm^3^, and the tumors, along with the livers, spleens and kidneys, were collected and weighed.

For animal models involving co-administered drugs, BALB/c mice were subcutaneously inoculated with Renca cells (1*10^6^ cells). When the average tumor volume reached approximately 100 mm^3^, the mice were randomly divided into 6 groups (n=5). The mice were treated with various agents, including curcumin (Solarbio, China), anti-PD-L1 (BioXcell, USA), anti-CTLA-4 (BioXcell, USA), and mouse IgG (control; BioXcell, USA). The specific groupings and treatment protocols are shown in **Figure 8**A.

### 4.4 Western blotting

Cells were lysed with RIPA buffer (Beyotime, China) supplemented with a proteinase and phosphatase inhibitor cocktail (Beyotime, USA). Protein concentration was measured using a BCA assay kit (Beyotime, China). Proteins were separated by 10% SDS-PAGE gels, transferred to polyvinylidene fluoride (PVDF) membranes (Millipore, USA), and visualized via western blotting with specific antibodies. The blots were captured using a gel image analysis system. The primary antibodies utilized in this investigation are enumerated in Table S3 (Supporting Information).

### 4.5 Co-immunoprecipitation (co-IP)

The cells were lysed in IP/Western lysis buffer (Beyotime, China) containing a proteinase and phosphatase inhibitor cocktail (Beyotime, USA). The protein A/G magnetic beads (Beyotime, China) were coincubated with anti-PTPN22, anti-CBL or anti-PD-L1 antibodies at 4 °C overnight. The cell lysates were incubated with protein A/G magnetic beads in a rotating incubator at 4 °C for 6 hours. The beads were washed with PBST and boiled in 2×SDS loading buffer for 10 minutes. Afterward, the beads were then removed by centrifugation, and the precipitated samples were analyzed by immunoblotting.

For lysis for two rounds IP, treated RCC cells were lysed in IP/Western lysis buffer containing a proteinase and phosphatase inhibitor cocktail. The cell lysates were incubated with anti-Flag agarose in a rotating incubator for 6 hours at 4 °C. After elution, the left complex was performed the 2nd immunoprecipitation (IP) with anti-Myc. Finally, the beads were washed with PBST and boiled in 2×SDS loading buffer for 10 minutes.

### 4.6 Histology and immunohistochemistry (IHC)

Tissues were embedded in paraffin and sectioned at a thickness of 5 µm. The sections were were deparaffinized in xylene and rehydrated using an ethanol gradient. Next, the sections were then subjected to staining with H&E or were boiled in an ethylenediaminetetraacetic acid (EDTA) antigen retrieval solution at pH 9.0 for 15 min. Then sections were then treated with 3% hydrogen peroxide and 5% goat serum to block endogenous peroxidase activity and nonspecific antigen binding. Following this, the sections were washed with PBS and incubated with the primary antibody at 4 °C overnight. After three washes with PBS, the sections were incubated with horseradish peroxidase (HRP)-conjugated secondary antibody, followed by a chromogenic reaction using diaminobenzidine as the chromogen.

### 4.7 Immunofluorescence (IF)

Cells were fixed with 4% formaldehyde for 15 min. After permeabilizing the cell membrane with 0.1% Triton X-100 for 10 min (not applicable to membrane proteins), the cells were incubated with 5% BSA for 15 min at 37 °C. The primary antibodies were incubated overnight at 4 °C, and the secondary antibodies were incubated the following day at room temperature and protected from light for 1 h. The cells were washed with PBS, and 4',6-Diamidino-2-phenylindole dihydrochloride (DAPI) was applied for 5 min to stained the nuclei.

### 4.8 Multiplex immunofluorescence (mIF)

Multiplex immunofluorescence staining was performed using the Opal™ TSA-based system (Akoya Biosciences) in accordance with the manufacturer's instructions. Sequential staining was carried out using primary antibodies specific to target markers, followed by incubation with horseradish peroxidase (HRP)-conjugated secondary antibodies and application of fluorophore-labeled tyramide signal amplification (TSA) reagents. Antigen retrieval was repeated between staining cycles to remove bound antibodies, while preserving covalently attached fluorophores. Opal fluorophores (Opal 480, 520, 570, 620, and 690) were employed for up to five-plex staining, and DAPI was used for nuclear counterstaining. The primary antibody panel included anti-CD3, anti-CD4, anti-CD8, anti-FOXP3, and anti-GZMB. Slides were scanned using the Vectra Polaris Automated Quantitative Pathology Imaging System (Akoya Biosciences) at 40× magnification. Spectral unmixing, tissue segmentation, cell phenotyping, and quantification of fluorescence intensity were performed using inForm image analysis software (Akoya Biosciences). Cell counts and marker co-expression statistics were further validated using ImageJ software.

### 4.9 PD-L1/PD-1 blockade assay

Renal cancer cells were subjected to various pretreatments. Subsequently, the culture medium was removed, and Jurkat cells stably transfected with PD-1 and the activated T-cell nuclear factor (NFAT) luciferase reporter were added together with a luciferase substrate. Co-culturing these cells leads to NFAT luciferase activation; however, the interaction between PD-L1 and PD-1 suppresses NFAT-driven luciferase activity. After 8 hours of co-culture, Bio-Glo reagent (Promega) was added to each well, and luminescence intensity was measured.

### 4.10 Total RNA isolation and quantitative real-time PCR (qRT-PCR)

Total RNA was extracted from cells using RNAiso Plus (Takara, Dalian, China). Following to the manufacturer's instructions, 1 μg RNA was reverse transcribed into cDNA using the Prime Foot RT Reagent Kit (Takara, Dalian, China). qRT-PCR was performed using a LightCycler® 96 Instrument (Roche, Basel, Switzerland) and results were normalized to the β-actin gene expression levels. Relative gene expression data were analyzed using the 2^-ΔΔCT^ method.

### 4.11 Flow cytometry

To measure the level of PD-L1 expression, cells were harvested, and the conjugated antibody was added to stain the cells on ice for 30 min. Subsequently, the expression of PD-L1 on cancer cells were detected using CytoFLEX flow cytometry (Beckman Coulter, CA, USA).

To quantification of tumor-infiltrating lymphocytes in subcutaneous tumor, tumor tissues from tumor-bearing mice were aseptically excised, cut into small pieces, and ground to obtain single-cell suspensions. The suspensions were filtered through a 70 μm strainer, and lymphocytes were isolated using density gradient centrifugation at 800 g for 30 minutes. The lymphocyte layer was collected, washed, and resuspended in PBS for downstream analysis. For surface marker staining, 10^6^ cells per sample were incubated with anti-CD45, anti-CD3, anti-CD4, anti-CD8 and anti-CD25 antibodies at 4 °C in the dark for 30 minutes. The cells were then washed, resuspended, and analyzed by flow cytometry. For intracellular staining, fixed and permeabilized cells were incubated with anti-GZMB and anti-FOXP3 antibodies at 4 °C in the dark for 60 minutes. After washing, the cells were analyzed by flow cytometry to assess lymphocyte proportions and functional marker expression.

### 4.12 Dephosphorylation of cell lysates

For dephosphorylation experiments, total proteins were extracted in RIPA lysis buffer, diluted to 1.0 mg/ml, and incubated with 20 U/μl λ-phosphatase in MnCl2 and enzyme buffer at 30 ℃ for 3 h. The reaction was stopped by sample buffer addition and heating to 95 °C for 5 min. Control samples were treated identically without λ-phosphatase.

### 4.13 Drug affinity responsive target (DARTS)

The DARTS assay was conducted with slight modification based on a previous description 43. Different concentrations of Curcumin or solvent control (DMSO) was added to the cell lysate and incubated for 1 hour at room temperature. Following this, the samples were digested at room temperature for 30 min with same proportions of enzyme. After stopping digestion, loading buffer was added, and the samples were boiled for Western blot analysis.

### 4.14 Cellular thermal shift assay (CETSA)

The CETSA assay was conducted according to a previous description 44. Cells were lysed with liquid nitrogen, the lysate samples were centrifuged, and the supernatant was collected. Curcumin (200 μM) or the solvent control (DMSO) was added and incubated at room temperature for 15 min. The sample was divided into 100 μL/tube, heated within a certain temperature range for 3 min, cooled at room temperature for 3 min, and stored on ice. All samples were centrifuged, and the supernatant was analyzed by Western blot.

### 4.15 Microscale thermophoresis (MST)

To verify the ability of Curcumin to bind PTPN22, we constructed HEK-293T cells with PTPN22-GFP, and cell lysates were obtained 48 h later. This was then tested with MonolithTM NT.115 MST equipment (Nano temper, Germany).

### 4.16 LC-MS/MS proteomics analysis

LC-MS/MS were performed by Shanghai OE Biotech. Co., Ltd. (China). Total protein was extracted from the samples, with a portion reserved for protein concentration measurement and SDS-PAGE, while the remaining portion was trypsinized. The digested peptides were desalted, and the samples were subsequently identified by LC-MS/MS. LC-MS/MS identification was performed by using Data independent acquisition (DIA) to collect the mass spectral data of each sample, enabling spectral matching, quantitative information extraction, and the subsequent statistical analyses.

### 4.17 Statistical analysis

All data were presented as mean±standard deviation (SD) unless otherwise noted. Statistical comparisons between the two groups were conducted using a two-tailed unpaired Student's t-test. For comparisons among three or more groups, a one-way analysis of variance (ANOVA) followed by appropriate post hoc tests was applied. Dose-response curves and IC_50_ values were calculated using nonlinear regression analysis in GraphPad Prism (version 10.1.2; GraphPad Software Inc., San Diego, CA, USA). Correlations between variables were evaluated using Pearson's correlation coefficient. All statistical analyses were conducted using SPSS software (version 23.0; IBM Corp., Armonk, NY, USA) and GraphPad Prism. A p-value < 0.05 was considered statistically significant. A significance level of *p* < 0.05 was considered indicative of statistically significant. *p* values are reported as follows: * *p* < 0.05; ** *p* < 0.01 and *** *p* < 0.001.

## Supplementary Material

Supplementary figures and tables.

## Abbreviations

TME: tumor microenvironment
RCC: renal cell carcinoma
PTPN22: Protein tyrosine phosphatase N22
ICI: immune checkpoint inhibition
ccRCC: clear cell Renal cell carcinoma
RTKs: regulates receptor tyrosine kinases
Tyr: tyrosine
TCR: T cell antigen receptor
TCGA: Cancer Genome Atlas
KIRC: kidney renal clear cell carcinoma
TIDE: Tumor Immune Dysfunction and Rejection
IHC: Immunohistochemistry
IF: immunofluorescence
mIF: Multiplex Immunofluorescence
DEPs: differentially expressed proteins
PCA: principal-component analysis
irAEs: immune-related adverse events
TILs: tumor-infiltrating lymphocytes
co-IP: Co-Immunoprecipitation
qRT-PCR: Quantitative real-time PCR
DARTS: Drug affinity responsive target
CETSA: Cellular Thermal Shift Assay
MST: Microscale Thermophoresis
