# Supplementary material for: PTPN22 Dephosphorylates CBL to Inhibit PD-L1 Ubiquitination and Drive Immunosuppression in Renal Cell Carcinoma
Source: Int J Biol Sci. 2026 Jan 1;22(2):663–83. doi: 10.7150/ijbs.122418 (PMC12780949; doi:10.7150/ijbs.122418)
Supplement: Supplementary file 1 — Supplementary figures and tables. [file ijbsv22p0663s1.pdf]

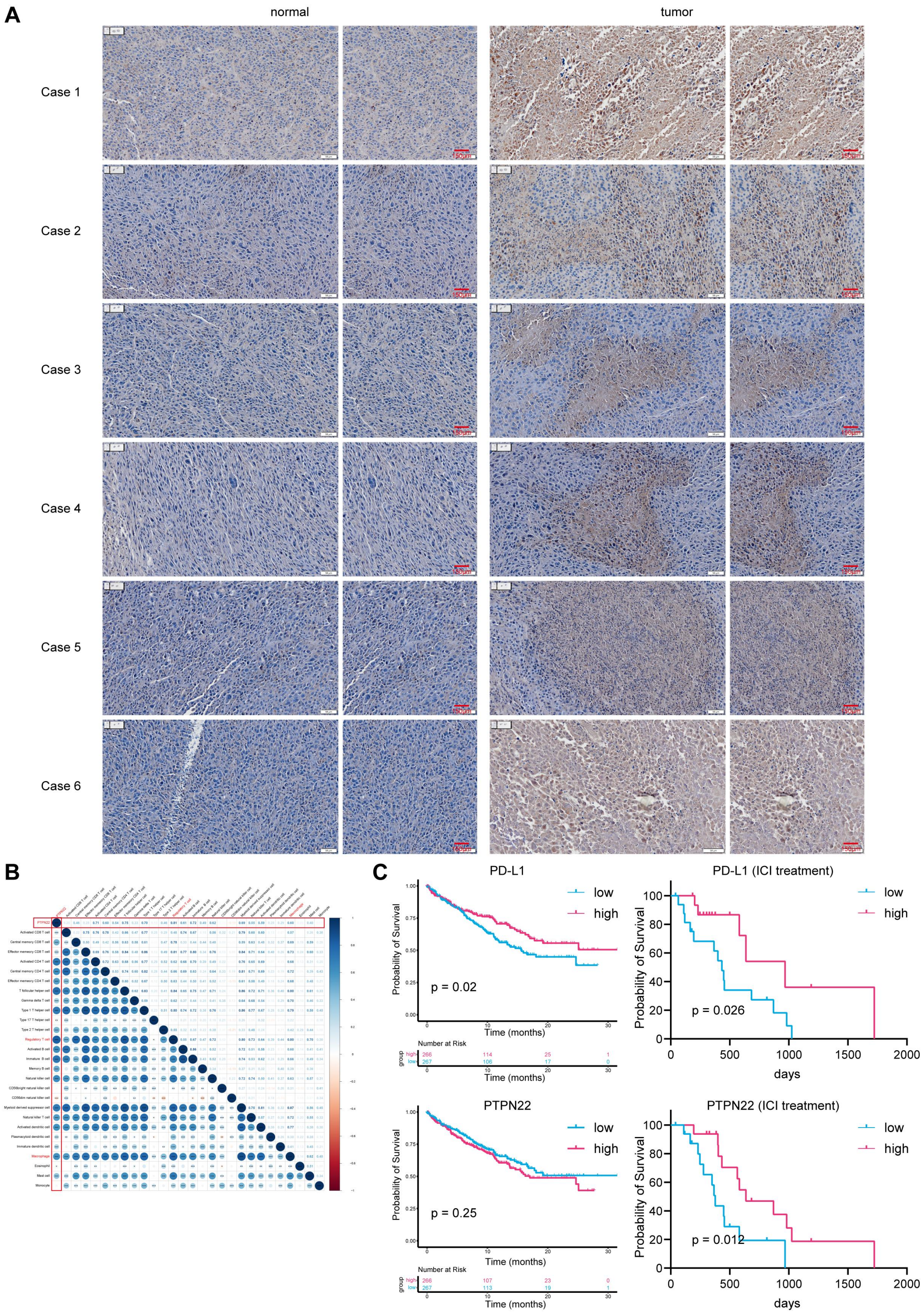

Figure S1. PTPN22 is overexpressed and is associated with immunosuppression in RCC. A) Representative IHC images for PTPN22 expression in normal and tumor tissues. Scale bar = 150 μm. B) Correlation heatmap of PTPN22 between differential immune fraction. C) The survival of RCC patients stratified by the expression of PTPN22 or PD-L1 was compared by two-sided log-rank analysis. ns, not significant; **p* < 0.05, ***p* < 0.01 and ****p* < 0.001.

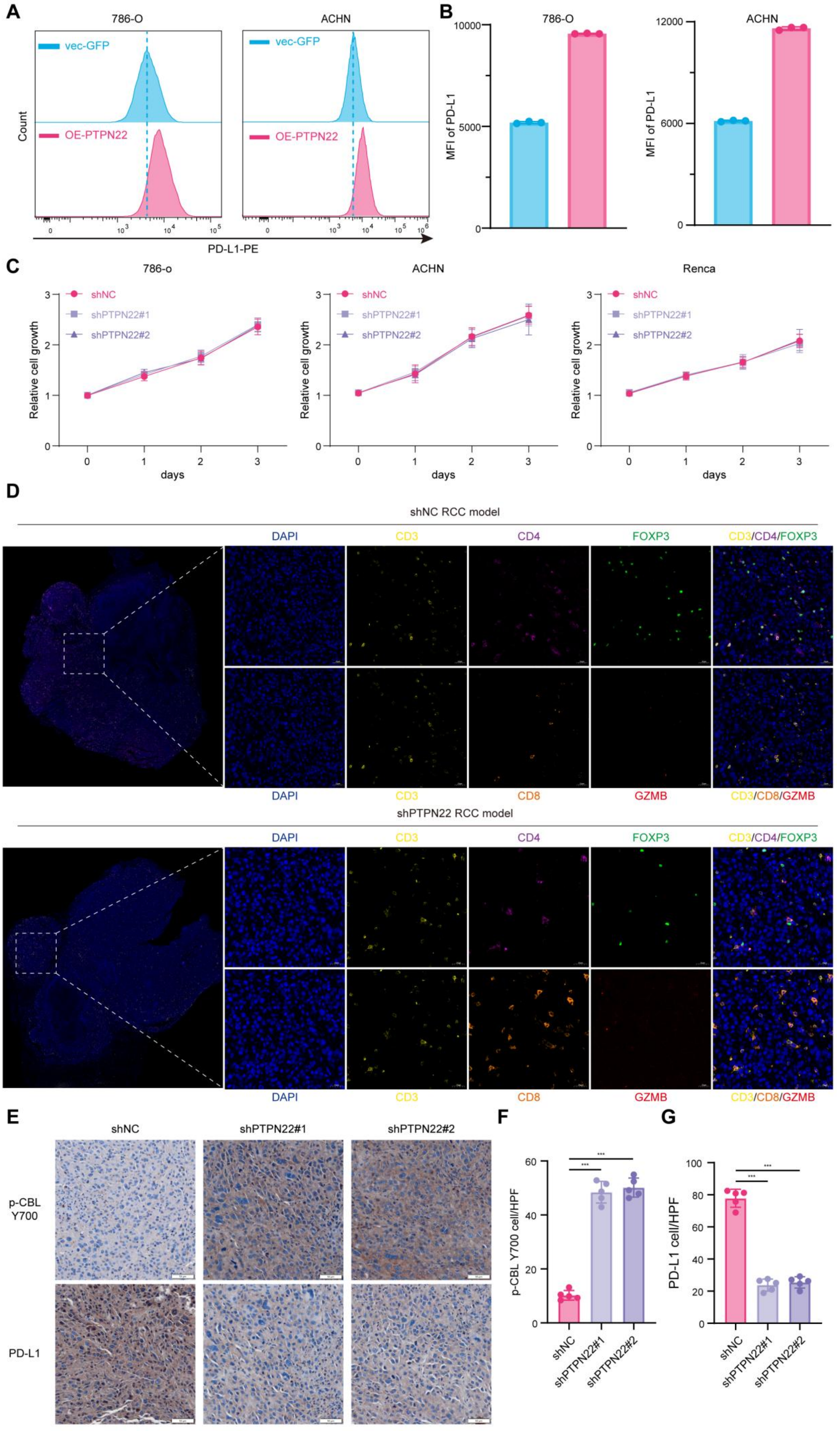

A
786-O
ACHN
vec-GFP
OE-PTPN22
Count
PD-L1-PE
B
MFI of PD-L1
C
786-o
Renca
shNC
shPTPN22#1
shPTPN22#2
Relative cell growth
days
D
shNC RCC model
shPTPN22 RCC model
DAPI
CD3
CD4
FOXP3
CD3/CD4/FOXP3
CD8
GZMB
CD3/CD8/GZMB
E
p-CBL Y700
PD-L1
F
p-CBL Y700 cell/HPF
G
PD-L1 cell/HPF

Figure S2. PTPN22 influences tumor growth by modulating T cell infiltration in the tumor immune microenvironment. A) Flow cytometry analysis of PD-L1 in 786-O and ACHN cells after PTPN22 overexpression. B) Statistical analysis of PD-L1 MFI. C) CCK8 analysis assays of RCC cells with or without shPTPN22. D) Representative images of mIF for DAPI (blue), CD3 (yellow), CD4 (purple), FOXP3 (green), CD8 (orange), GZMB (red). E-G) Representative IHC staining images (left panel) and quantification (right panel) for PD-L1 and p-CBL Y700 of BALB/c mice with indicated treatment. ns, not significant; *p < 0.05, **p < 0.01 and ***p < 0.001.

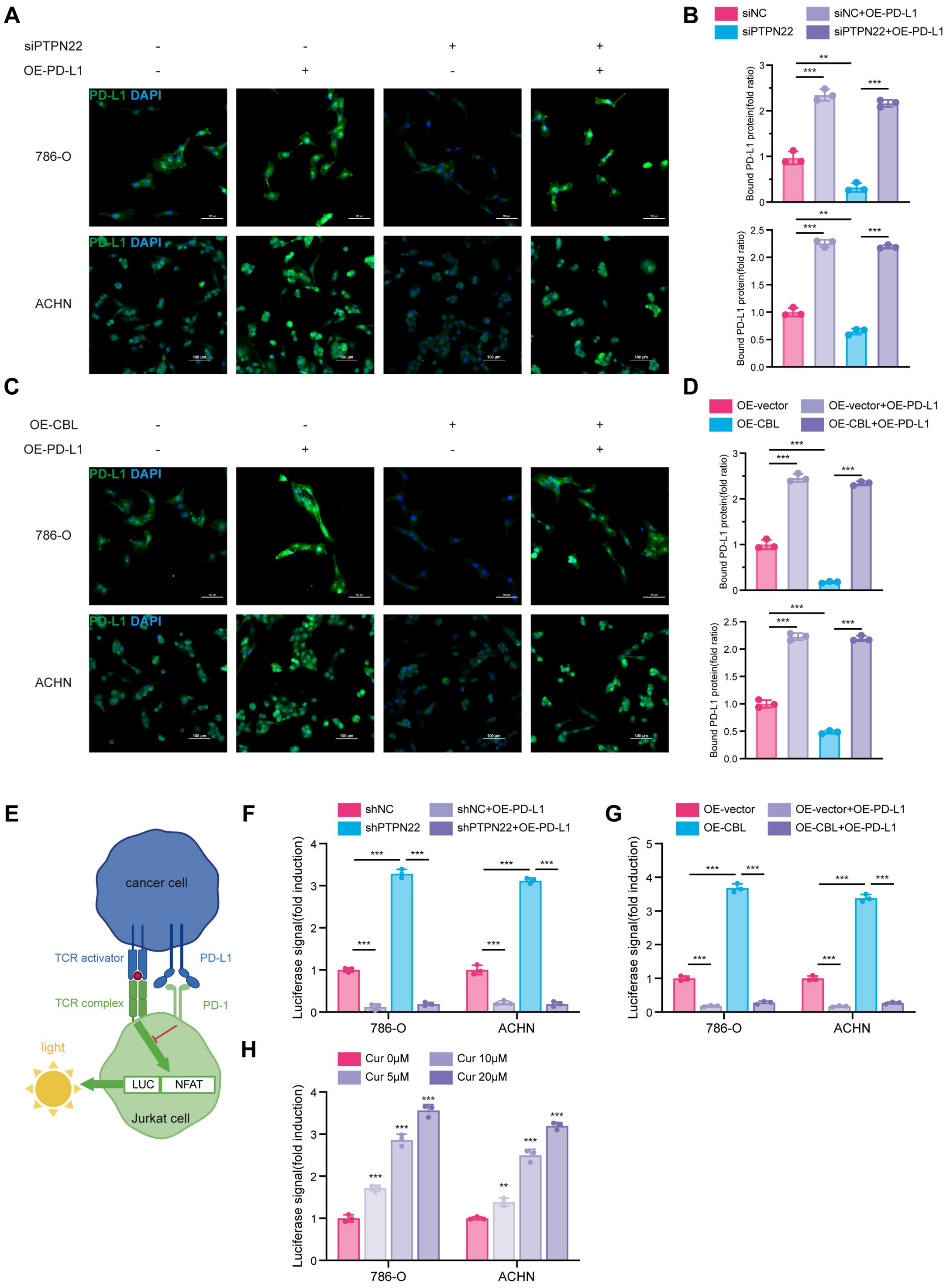

Figure S3. Silencing PTPN22 reduces the binding between tumor cells and PD-1, and promotes T cell activation. A, B) PD-L1/PD-1 binding assay (left panel) and quantification (right panel) in RCC cells with indicated treatment. C, D) PD-L1/PD-1 binding assay (left panel) and quantification (right panel) in RCC cells with indicated treatment. E) Schematic diagram of the PD-L1/PD-1 blockade assay. F-H) The statistical analysis of PD-L1/PD-1 blockade assays in different treatment groups. ns, not significant; *p < 0.05, **p < 0.01 and ***p < 0.001.

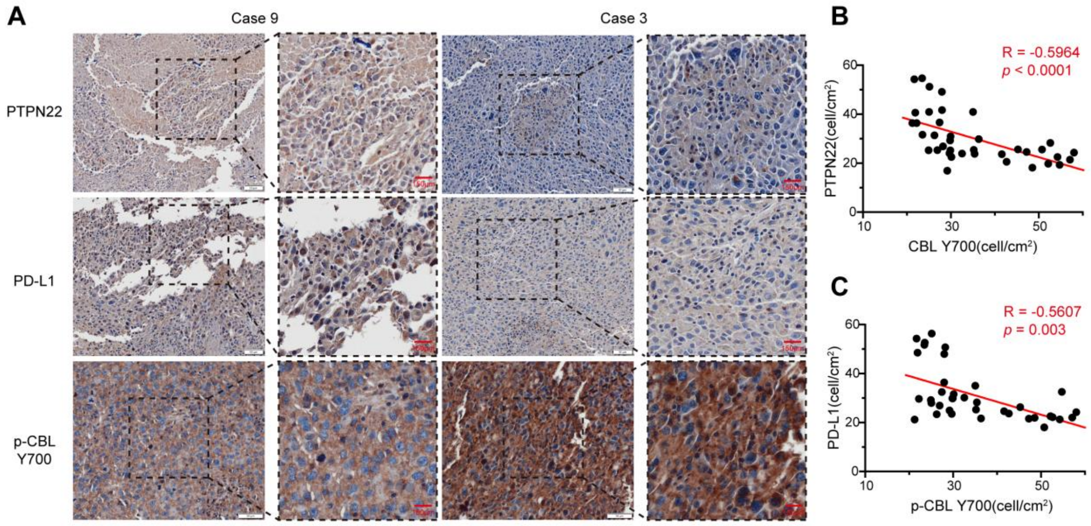

Figure S4. Validation of the correlation among PTPN22, p-CBL Y700, and PD-L1 in tumor tissues. A) Representative immunohistochemistry (IHC) images of PTPN22, PD-L1 and p-CBL Y700 expression in patient-derived tissues. B) Correlation analysis of PTPN22 and p-CBL Y700 protein levels in patient-derived tissues.

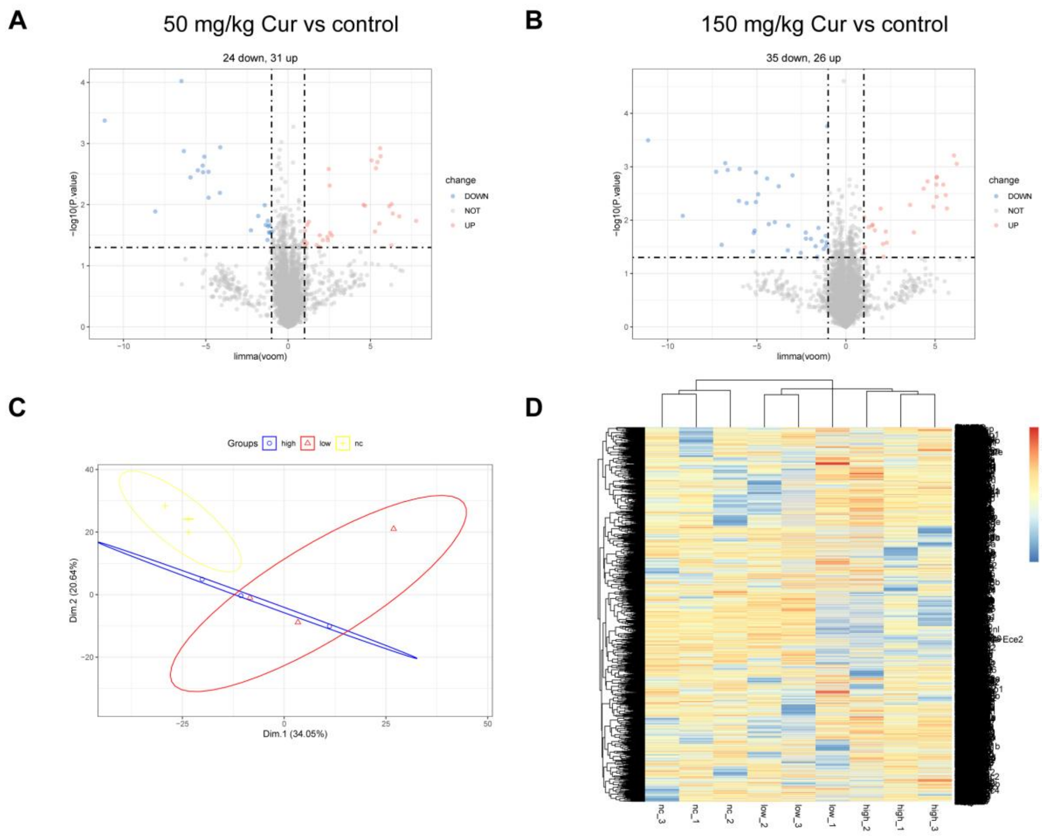

Figure S5. Proteomic analysis of Cur effects after treatment of Renca tumor in vivo. A) Volcano plot of DEPs analysis between 50 mg/kg Cur treatment with solvent control. B) Volcano plot of DEPs analysis between 150 mg/kg Cur treatment with solvent control. C) PCA showing the expression of proteins in Cur-treatmented tumors. D) Heatmap showing the expression of proteins in Cur-treatmented tumors.

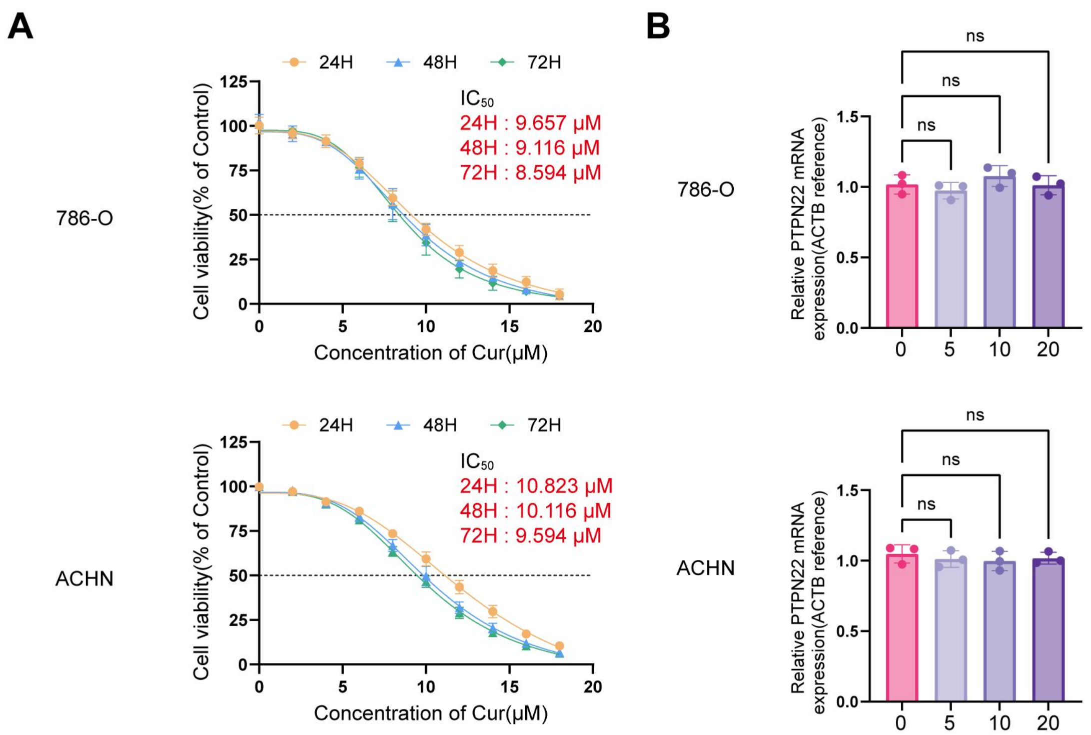

Figure S6. Curcumin promotes PTPN22 degradation in RCC cells. A) CCK-8 assay showing drug effects on cell viability. B) qRT-PCR showing relative PTPN22 RNA expression in 786-O and ACHN cells treated with different curcumin concentrations. ns, not significant; $*p < 0.05$, $**p < 0.01$ and $***p < 0.001$.

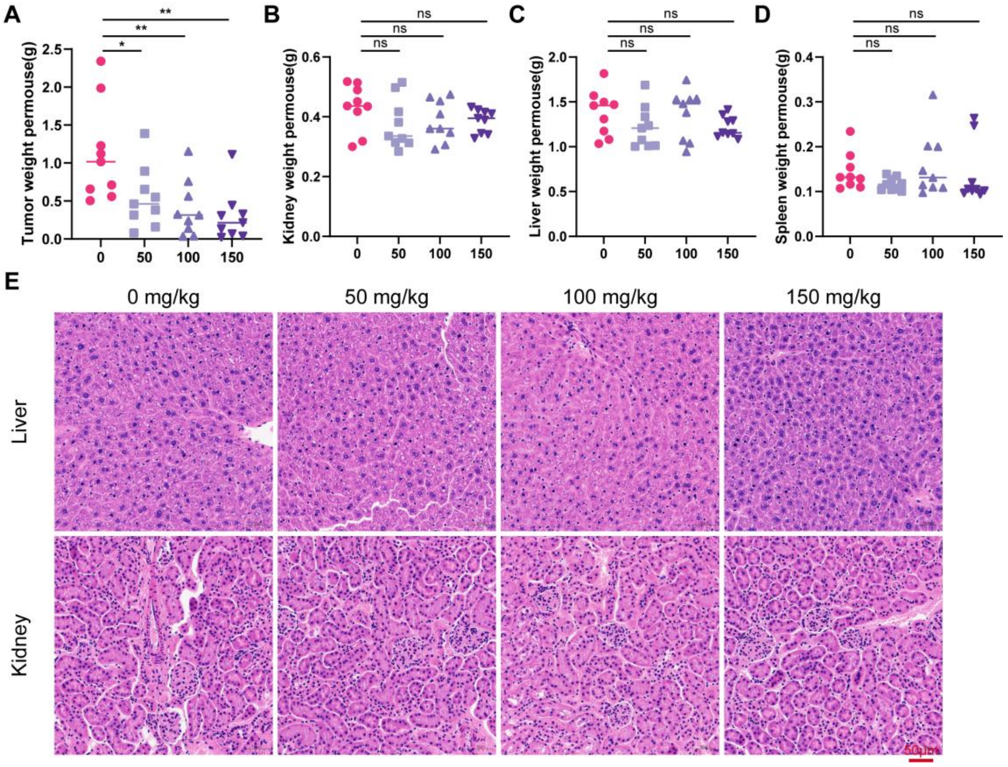

Figure S7. Curcumin's anti-tumor effects and safety. A-D) Tumor weight (A), kidney weight (B), liver weight (C) and spleen weight (D) of BALB/c mice with indicated treatment. (n = 9 per group). E) Representative H&E images of BALB/c mice with indicated treatment. (n = 9 per group). ns, not significant; *p < 0.05, **p < 0.01 and ***p < 0.001.

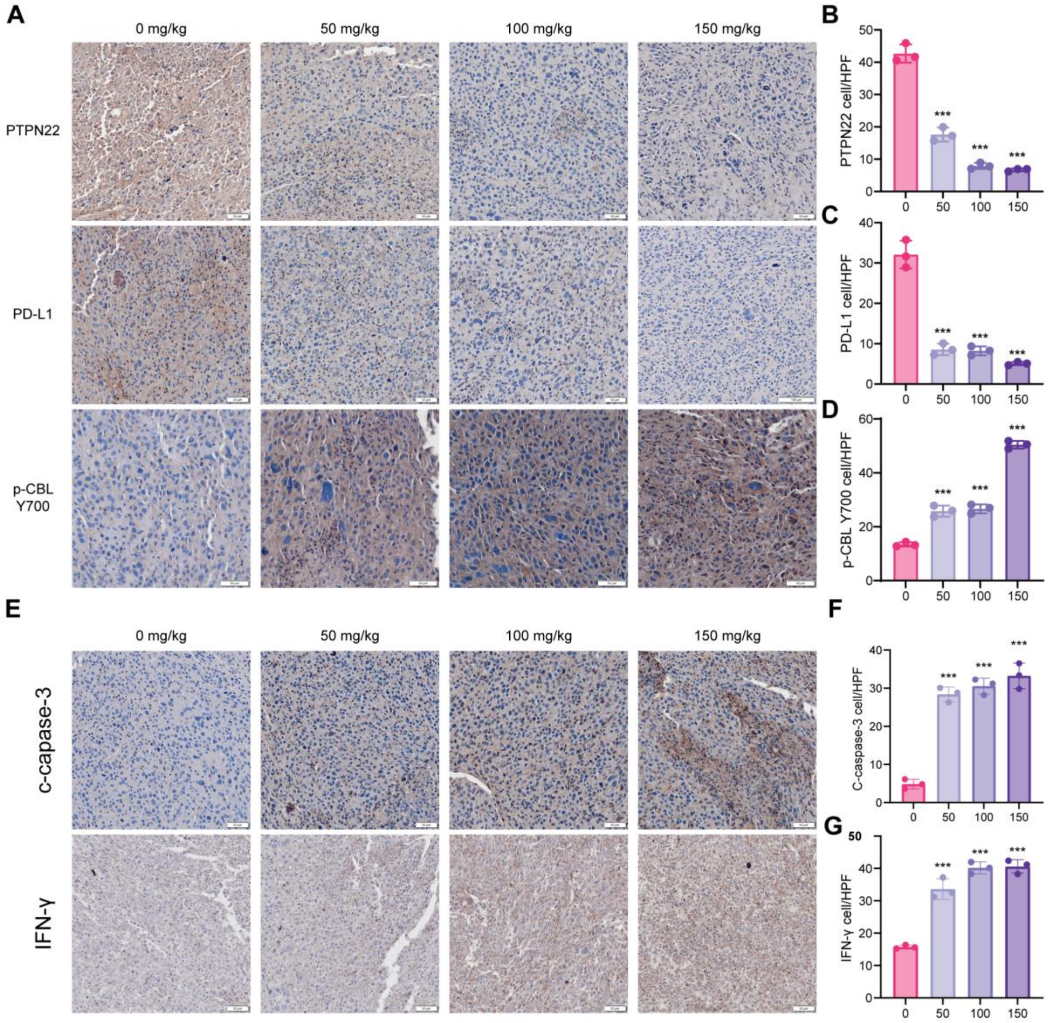

Figure S8. Curcumin effectively downregulates PTPN22 and PD-L1 and promotes tumor apoptosis and immunocide. A) Representative IHC staining images (left panel) and quantification (right panel) for PTPN22 and PD-L1 of BALB/c mice with indicated treatment. B) Representative IHC staining images (left panel) and quantification (right panel) for c-caspase-3 and INF-γ of BALB/c mice with indicated treatment. ns, not significant; *$p < 0.05$, **$p < 0.01$ and ***$p < 0.001$.

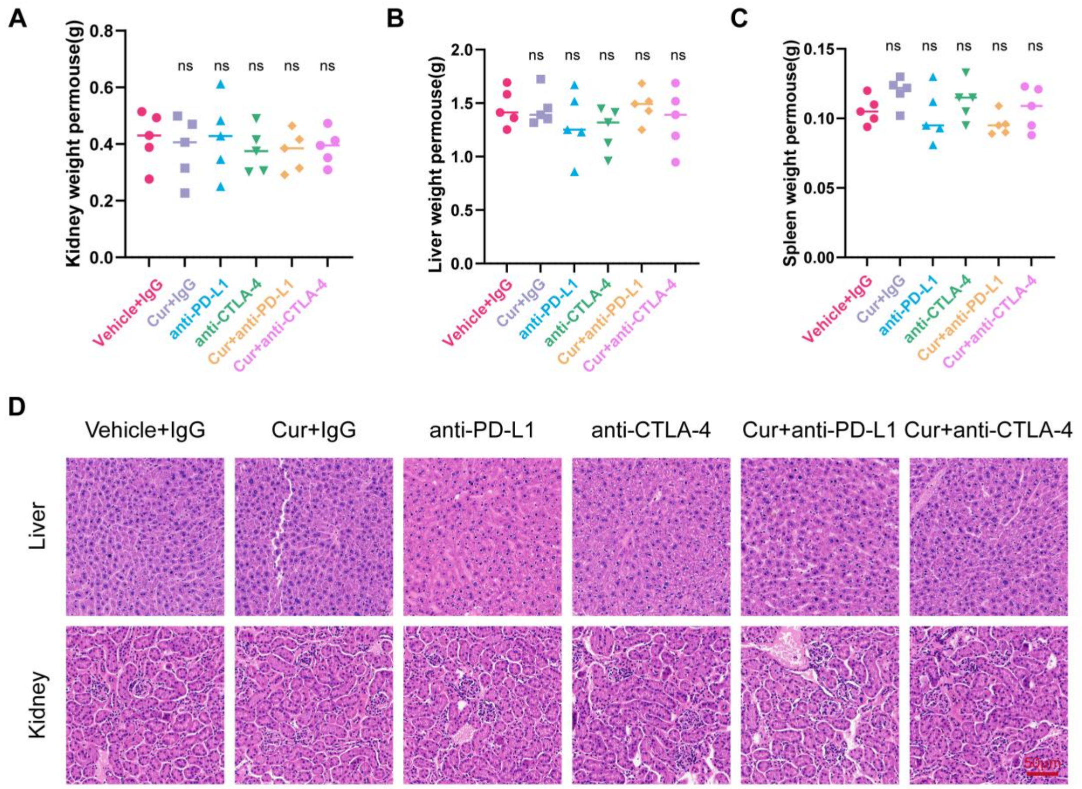

Figure S9. Curcumin in combination with ICIs does not cause systemic toxicity in mice. A,B,C) kidney weight (A), liver weight (B) and spleen weight (C) of BALB/c mice with indicated treatment. (n = 5 per group). D) Representative H&E images of BALB/c mice with indicated treatment. (n = 5 per group). ns, not significant; *p < 0.05, **p < 0.01 and ***p < 0.001.

Table S1: Clinical characteristics of renal cell carcinoma patient's cohort and applications

Name	Sex	Location	Diagnosis	Staging	Primary or Recurrence	Application
M051	Male	Left Kidney	ccRCC	$T_2N_0M_0$	Primary	IHC
M074	Female	Left Kidney	ccRCC	$T_1N_0M_0$	Primary	IHC
M065	Female	Right Kidney	ccRCC	$T_3N_1M_0$	Primary	IHC
M103	Female	Left Kidney	ccRCC	$T_1N_1M_0$	Primary	IHC
M110	Female	Left Kidney	ccRCC	$T_1N_0M_0$	Primary	IHC
M123	Female	Right Kidney	ccRCC	$T_3N_0M_0$	Primary	IHC
M137	Male	Left Kidney	ccRCC	$T_2N_1M_0$	Primary	IHC
M157	Female	Left Kidney	ccRCC	$T_3N_0M_0$	Primary	IHC
M217	Male	Right Kidney	ccRCC	$T_3N_0M_0$	Primary	IHC
M216	Male	Right Kidney	ccRCC	$T_2N_1M_0$	Primary	IHC
M299	Male	Right Kidney	ccRCC	$T_1N_0M_0$	Primary	IHC
R007	Male	Left Kidney	ccRCC	$T_1N_0M_0$	Primary	IHC
R055	Female	Right Kidney	ccRCC	$T_1N_0M_0$	Primary	IHC
R052	Male	Left Kidney	ccRCC	$T_3N_1M_0$	Primary	IHC

Table S2: Sequences of shRNAs in this study and primers for quantitative real-time PCR

Name	Target Sequence	Source
shPTPN22#1	5'-GTACGGACACCTGAATCATTT-3'	TranSheepBio,Shanghai,China
shPTPN22#2	5'-GAATTGATACAGCAGAGAGAA-3'	TranSheepBio,Shanghai,China
shCBL#1	5'-GTACGTATGAAGCAATGTATA-3'	TranSheepBio,Shanghai,China
shCBL#2	5'-GATTCTCCGAACGTGTCACGT-3'	TranSheepBio,Shanghai,China

rtPCR primers	Sequence(5' to 3')
PD-L1 Forward	TGGCATTTGCTGAACGCATTT
PD-L1 Reverse	TGCAGCCAGGTCTAATTGTTTT
PTPN22 Forward	TGCTACCCAGGGTCCTTTATC
PTPN22 Reverse	AACTCCATACATGCCATGACAAT
ACTB Forward	CATGTACGTTGCTATCCAGGC
ACTB Reverse	CTCCTTAATGTCACGCACGAT

Table S3. Antibodies used in this study

Antibodies	Company	Cat no.
GAPDH	Affinity	AF7021
PTPN22	Abclonal	A1406
PD-L1	Proteintech	28076-1-AP
PD-L1	Huabio	HA721176
PD-L1	Huabio	HA722184
PD-L1-FITC	Abclonal	A22304
PD-L1-mCherry	Abclonal	A24570
UB	Huabio	ET1609-21
CBL	Abclonal	A0732
Pan Phospho-Tyrosine	Abclonal	AP1162
Phospho-CBL (Tyr647)	Affinity	AF3225
Phospho-CBL (Tyr700)	Abclonal	AP0780
Phospho-CBL (Tyr731)	Affinity	AF8006
Phospho-CBL (Tyr774)	Abclonal	AP0794
K48-UB	CST	4289
K63-UB	CST	5621
FLAG	Huabio	HA722780
His	Huabio	HA722798
Myc	Huabio	R1208-1
HA	Huabio	ET1611-49
PSMB5	Proteintech	19178-1-AP
FOXP3	Abclonal	A27744
GZMB	Abclonal	A2557
c-capase-3	Huabio	ET1608-64
IFN-γ	Proteintech	30293-1-AP
L/D,FVS780	BD Pharmingen	565388
CD45,FITC	BD Pharmingen	553079
CD3,PE	BD Pharmingen	553063
CD8,R718	BD Pharmingen	566985
CD4,APC	BD Pharmingen	553051
CD25,PE-CY7	BD Pharmingen	552880
FOXP3,BV421	Thermo	404-5773-82
GZMB,BV785	Biolegend	396438

Table S4. 114 traditional Chinese medicine monomers

Betulinic acid	Cepharanthine	18 β-Glycyrrhetinic acid	(E)-Ethyl p-methoxycinnamate	β-Elemonic acid
Chelerythrine (chloride)	Ayanin	Fisetin	Columbianadin	Hydroxytyrosol
Aloperine	Osthole	4'-Hydroxychalcone	18α-Glycyrrhetinic acid	Fraxetin
Lupenone	Chlorogenic acid	Asiatic acid	Liquiritin	Terpinen-4-ol
Zingerone	Macelignan	1,4-Cineole	Liquiritigenin	Morusin
Sinomenine	Solasodine	Avicularin	Glabridin	Carnosic acid
Sinomenine hydrochloride	Sarsasapogenin	Fraxinellone	Decursinol	Aucubin
Resveratrol	Naringenin	Aloesin	Wogonin	Quercetagetin
Genipin	Isoliquiritigenin	Cordycepin	4(3H)-Quinazolinone	trans-Chalcone
Decursin	Sophocarpine	Ligustrazine	Sophoricoside	Coumarin
Triptonide	Sophocarpine (monohydrate)	Caffeic acid phenethyl ester	Licochalcone D	Carvacrol
Melatonin	Rhein	Colcemid	Acacetin	8-O-Acetylharpagide
Sinapine	Salicin	Esculetin	(-)-Alkannin	Isoorientin
(-)-Epicatechin	Nobiletin	Lycorine	Britannin	Isopimpinellin
Oridonin	Oxymatrine	Lycorine (hydrochloride)	6-Hydroxycoumarin	1-beta-D-Arabinofuranosyluracil
Baohuoside I	Luteolin	Ginsenoside Rk1	Nitidine (chloride)	Isorhamnetin
Bergenin	Magnolol	Ziyuglycoside II	Gallic acid (hydrate)	Camphor
Diphyllin	Beta-Sitosterol (purity>80%)	Farrerol	Allantoin	Abietic acid
Arctigenin	Beta-Sitosterol	Pseudolaric Acid B	Wedelolactone	Catalpol
Pterostilbene	Ailanthone	Lapachol	Salvigenin	Rhapontigenin
Jaceosidin	Artemisic acid	Isorhapontigenin	Crebanine	4-Hydroxybenzyl alcohol

Curcumin	Withaferin A	Methyl 3,4-dihydroxybenzoate	Curcumenol	Betulonic acid
Benzothiazole	Acetylshikonin	Citric acid	Isoeugenol acetate	

Table S5. The prediction performance of 114 traditional Chinese medicine monomers docked with PTPN22 protein

monomers	Mean Vina	monomers	Mean Vina
18-β-Glycyrrhetinic_acid	-8.62	Chlorogenic_acid	-7.23
Solasodine	-8.25	Ailanthone	-7.23
Curcumin	-8.23	Lupenone	-7.20
Withaferin_A	-8.11	Betulonic_acid	-7.17
Cepharanthine	-7.52	Diphyllin	-7.13
Sarsasapogenin	-7.52	Quercetagetin	-7.10
18-α-Glycyrrhetinic_acid	-7.46	Luteolin	-7.10
Ziyuglycoside_II	-7.32	Betulinic_acid	-7.07
Liquiritin	-7.30	Glabridin	-7.07
Morusin	-7.27	Sophoricoside	-7.07
